# Isolated Pancreatic Metastases of Renal Cell Carcinoma—Clinical Particularities and Seed and Soil Hypothesis

**DOI:** 10.3390/cancers15020339

**Published:** 2023-01-04

**Authors:** Franz Sellner, Sabine Thalhammer, Martin Klimpfinger

**Affiliations:** 1 Department of General, Visceral and Vascular Surgery, Clinic Favoriten, Kaiser Franz Josef Hospital, 1100 Vienna, Austria; 2 Clinical Institute of Pathology, Medical University, 1090 Vienna, Austria

**Keywords:** renal cell carcinoma, pancreatic metastasis, risk-factors, seed and soil mechanism

## Abstract

**Simple Summary:**

A meta-analysis of 1470 isolated pancreatic metastases of renal cell carcinoma revealed that these are characterised by not only the exclusive occurrence of PM and a good prognosis but a lack of prognostic significance of volume and growth rate dependent risk factors and the independence of treatment results from standard or local resections. The good prognosis affects not only isolated PM, but also multi-organ metastases of the RCC, in which the additional occurrence of PM is also associated with a better prognosis. As an explanation for all these peculiarities, a strong acting seed and soil mechanism can serve, which allows metastases settlement only in the pancreas, but prevents them definitively or for years in all other organs. Genetic studies revealed specific changes in cases of PM of RCC and a lack of loss of 9p and 14q, which are otherwise specific gene mutations at the onset of generalization, a low weight genome instability index, and a low rate of PAB1 and a high rate of BPRM1 alterations, which signal a more favourable course. The cause of pancreatic organotropism in isPMRCC, however this is still unclear.

**Abstract:**

A meta-analysis of 1470 isolated pancreatic metastases of renal cell carcinoma revealed, that, in addition to the unusual exclusive occurrence of pancreatic metastases and the favourable treatment results, the isPMRCC is characterised by further peculiarities of the clinical course: The lack of prognostic significance of volume and growth rate dependent risk factors and the independence of treatment results from standard or local resections. As an explanation for all these peculiarities, according to today’s knowledge, a strong acting seed and soil mechanism can serve, which allows embolized tumour cells to grow to metastases only in the pancreas, and prevents them definitively or for years in all other organs. The good prognosis affects not only isolated PM, but also multi-organ metastases of the RCC, in which the additional occurrence of PM is also associated with a better prognosis. Genetic studies revealed specific changes in cases of PM of RCC: Lack of loss of 9p21.3 and 14q31.2, which are otherwise specific gene mutations at the onset of generalization, a low weight genome instability index, i.e., high genetic stability, and a low rate of PAB1 and a high rate of BPRM1 alterations, which signal a more favourable course. The cause of pancreatic organotropism in isPMRCC is still unclear, so only those factors that have been identified as promoting organotropism in other, more frequent tumour entities can be presented: Formation of the pre-metastatic niche, chemokine receptor–ligand mechanism, ability to metabolic adaptation, and immune surveillance.

## 1. Introduction

Isolated pancreatic metastases (isPM) are a rare and unusual metastasis course of renal cell cancer (RCC), in which metastases occur only and exclusively in the pancreas, either definitively or over a long period of time. In addition, studies in recent years have shown that there are still other peculiarities of the clinical course in this entity that are not otherwise observed in metastasizing RCC. In addition to the extraordinarily favourable outcome, these features include the lack of prognostic significance of tumour volume and tumour growth rate dependent risk factors and the lack of influence of the extent of surgical therapy (local versus standard resections). Based on a comprehensive literature review with meta-analysis, the present study provides an overview of the totality of these characteristics, presents and analyses the present data and discusses the possible relationship of these characteristics to the seed and soil mechanism (SSM). 

## 2. Material and Methodology

A systematic review was performed following PRISMA guidelines. The literature data were obtained from the MEDLINE (PubMed) database from 1952 (first description of an isPM [1]) to 1 October 2022. The search criteria were: Renal cell carcinoma and pancreatic metastasis. Inclusion criteria were casuistic reports and single and multicentre institutional reports about isPM that occurred synchronously or metachronous to the RCC diagnosis and did not have additional organ metastases at the time of isPM diagnosis or within 6 months before or after isPM diagnosis and which were termed isolated pancreatic metastases of RCC (isPMRCC). In order to minimize the potential risk inherent in analysing small numbers of cases, only those single and multicentre institutional reports that included 20 or more cases were included in the results. These works are called high volume studies. Exclusion criteria, and vice versa, were non-RCC pancreatis metastases, observations with missing histology and widespread metastatic disease. In isPMRCC reports all observations in which other organ metastases were present in addition to the PM or had occurred within 6 months before or after the isPM diagnosis were excluded from the examination. In the few cases where an institution published its results several times over the years (Johns Hopkins University School of Medicine; Verona University of Verona Hospital Trust; Dept. of Surgery University of Heidelberg; Dept. of Surgery, Memorial Sloan-Kettering Cancer Institute), the most detailed report was accepted for the analysis and the others excluded.

**Figure 1:**
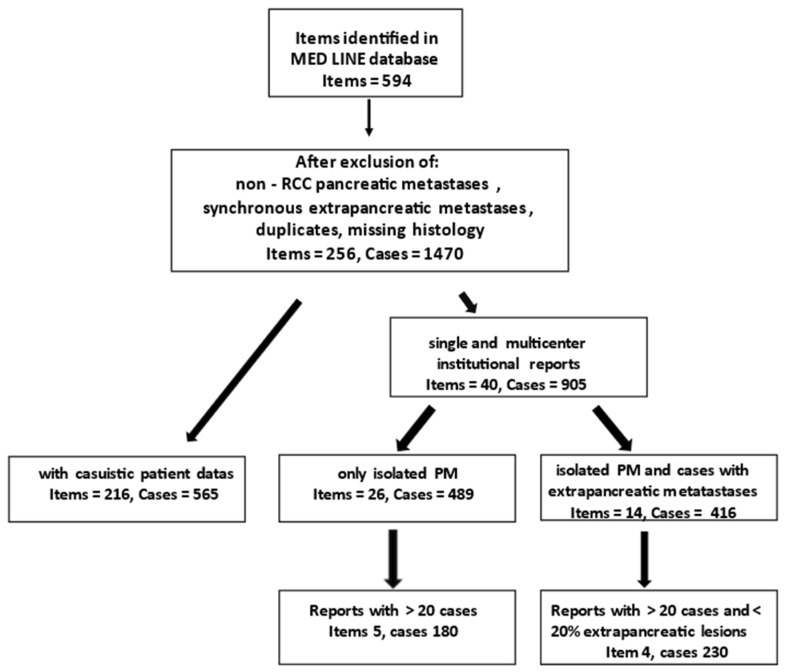
Search and selection strategy.

In addition to publications that are limited exclusively to isolated pancreatic metastases, there are also several papers that included isolated PM as well as individual cases of PM with synchronous-resectable, extrapancreatic metastases in their investigations if an R0 situation could be achieved and analysed in the two groups together as one collective. However, this is methodologically problematic for the questions raised here, since it is not certain that both groups exhibit identical clinical behaviour. The inclusion of this works in the isolated PM group of the RCC was therefore refrained from. In order not to lose completely the information contained in these publications, these reports are recorded and analysed as a separate group (“PM with synchronously resected extrapancreatic metastases”). However, to also limit the influence of extrapancreatic metastases on the results to a reasonable level in this group, all those publications were excluded in which the proportion of additional extrapancreatic lesions was >20%. In this way, a total of 1470 isPMRCC observations were compiled [1,2,3,4,5,6,7,8,9,10,11,12,13,14,15,16,17,18,19,20,21,22,23,24,25,26,27,28,29,30,31,32,33,34,35,36,37,38,39,40,41,42,43,44,45,46,47,48,49,50,51,52,53,54,55,56,57,58,59,60,61,62,63,64,65,66,67,68,69,70,71,72,73,74,75,76,77,78,79,80,81,82,83,84,85,86,87,88,89,90,91,92,93,94,95,96,97,98,99,100,101,102,103,104,105,106,107,108,109,110,111,112,113,114,115,116,117,118,119,120,121,122,123,124,125,126,127,128,129,130,131,132,133,134,135,136,137,138,139,140,141,142,143,144,145,146,147,148,149,150,151,152,153,154,155,156,157,158,159,160,161,162,163,164,165,166,167,168,169,170,171,172,173,174,175,176,177,178,179,180,181,182,183,184,185,186,187,188,189,190,191,192,193,194,195,196,197,198,199,200,201,202,203,204,205,206,207,208,209,210,211,212,213,214,215,216,217,218,219,220,221,222,223,224,225,226,227,228,229,230,231,232,233,234,235,236,237,238,239,240,241,242,243,244,245,246,247,248,249,250,251,252,253,254] (isolated PM of the RCC 565 casuistic observations, 489 in single and multicentre reports and 416 isPM in publications where in some cases additional synchronously resected extrapancreatic metastases existed), which form the basis of a meta-analysis (Figure 1).

In these groups, the influence of tumour volume-related risk factors (singular versus multiple PM, number and size of PM), growth rate dependent risk factors (synchronous versus metachronous PM, interval between RCC treatment and PM diagnosis) and extent of surgery on outcome was determined. It is in the nature of a retrospective study that not all relevant study details were provided in all cases included. The actual number of cases which can be used for each analysis is therefore given in each case. An analogous approach was taken when calculating the influence of time to metastasis onset and metastasis size. Only singular metastases were used in the calculations of metastases size. Standard resections were partial DP, distal pancreatectomy and total DP; local resections were operations described as metastasis enucleation/resection/extirpation and midsegment resection.

### Statistics

Kaplan-Meier curves were plotted with differences between strata determined by log rank tests. A Cox proportional hazard regression analysis was applied to determine the influence of possible risk factors on survival, such as the number and diameter of metastasis and the time interval until the occurrence of isPMRCC. Hazard ratio and their 95% confidence intervals (IV) were reported. A *p*-value < 0.05 was considered statistically significant. Statistical analyses were performed with SPSS, version 25.0 (SPSS, Chicago, IL, USA).

## 3. Results

### 3.1. TreatmentOoutcomes

#### 3.1.1. Surgical Therapy

**Figure 2:**
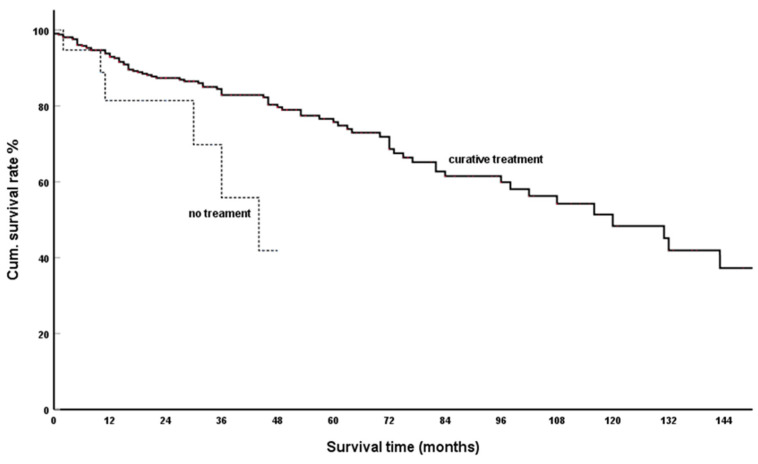
Surgical treatment vs. non-treatment group; Kaplan-Meier survival curves (*p* = 0.013).

Depending on the location and number of PM as well as the general condition of the patient, this was performed as partial or total duodenopancreatectomy, distal pancreatic resection, central pancreatectomy and local tumour resection. From 421 cases reported casuistically, a cumulative 5- and 10-year survival rate (SR) of 75.7% and 48.4%, respectively, can be calculated (Figure 2). In single and multicentre institutional reports of isolated PM [99,143,183,185,191,201,209,216,234], comparable results were reported with five-year SR of 52%–84%, 53% [99], 61% [143], 52% [183], 72% [184], 56% [190], 71% [200], 69% [208], 80% [215], 84% (Median 72%) [233]. 10a: 63% (median 69%) [254]. This also applies to the reports that additionally included cases with synchronously resected extrapancreatic metastases in their analyses [134,176,181,191,195,211,229,232,235,239,242,243] 88% [134]; 77% [176]; 63% [181], 79% [191], 72% [195], 50% [211], 72% [232], 79% [235], 81% [239], 68% [242], 83% [243], 77% [229]. 10a Schwarz 32%, Milanetto 55%: five-year SR 50–88% (median 77%).

#### 3.1.2. Spontaneous Course of IsPMRCC

In Figure 2, we also present the results of the few observations that were not administered surgically or targeted therapy (TKI, m-tor inhibitors or immunotherapy). The three-year SR of 56% calculated from 19 casuistically reported observations [10,22,63,64,78,90,107,166,175,185,188,194,216,250] is a favourable result for the spontaneous course of a metastatic RCC. However, the result is significantly worse than after interventions with curative intent (*p* = 0.013).

#### 3.1.3. Systemic Treatment

Since the introduction of targeted therapies in the form of tyrosine kinase inhibitors (TKI), M-Tor inhibitors (MTI) and Immune Checkpoint Inhibitors (ICI) such as Anti PD1 and Anti PD1L and AntiCTLA4, effective drug therapy has been available in advanced RCC. These agents revolutionized the systemic treatment of mRCC and caused a significant prolongation of progression free survival and OS, which also affected the PM of the RCC [158,175,204,255,256,257,258]. Santoni et al. [189] compared TKI treatment with surgical treatment in a large retrospective multicentre study of 103 PM of the RCC with 95% isolated PM in the resected cases and 24% in the systemic treated patients. With a median OS of 103 months versus 86 months, the difference between resected and unresected patients was not significant in terms of overall survival (OS). The good response of the isPMRCC to TKI, however, as Singla et al. [238] was able to show, is contrasted with a refractoriness to ICI. Both results: the good response to TKI and the nonresponse to ICI could be explained by the enrichment of angiogenic markers and low levels of inflammatory biomarkers in PM observations [238].

### 3.2. Tumour Volume-Dependent Risk Factors

#### 3.2.1. Single Versus Multiple Metastases

**Figure 3:**
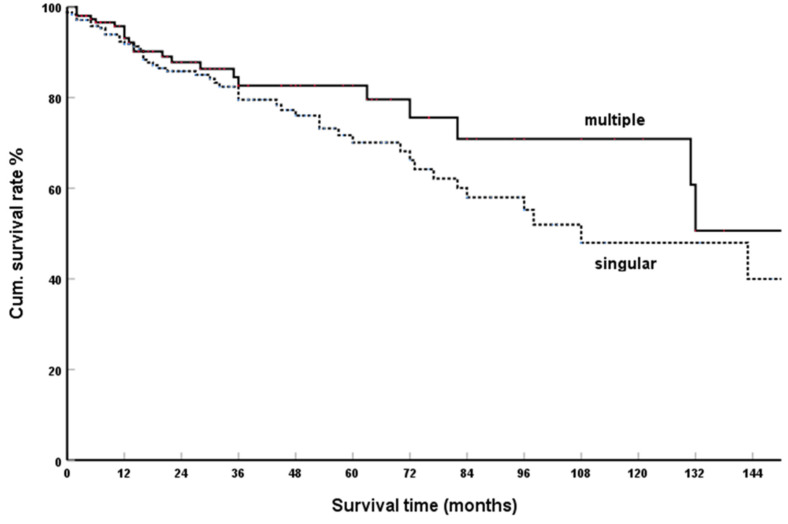
Solitary vs. multiple isPMRCC; Kaplan-Meier survival curves (*p* = 0.162).

From 396 sufficiently documented casuistic observations, a cumulative five-year SR of 70.4% and 79.6%, respectively, for single and multiple metastases was calculated (Figure 3). The difference in survival time is not significant (*p* = 0.162).

In high volume single and multicentre reports and literature reviews of isolated PM, it was also reported that single or multiple occurrence of PM has no significant influence on the OS: Konstantinidis et al. [143], N = 20, n. s. (*p* = 0.87); Tosoian et al. [183], N = 42, n. s. (*p* = 0.727); Benhaim et al. [184], N = 20, n. s. (*p* value not specified); Anderson et al. [215], N = 20, n. s. (*p* > 0.05); Malleo et al. [245], N = 69, n. s. (*p* = 0.77); Reddy et al. [259], N = 21, n. s. Similar results were found in the reports, which included cases of PM with synchronously resectable extrapancreatic metastases in addition to isolated PM: Schwarz et al. [181], N = 62, n. s. (*p* = 0.9); Di Franco et al. [232], N = 21, n. s. (*p* = 0.391); Milanetto et al. [235], N = 39, n.s. (*p* = 0.9); Blanco-Fernández et al. [243], N = 116, n. s. (*p* = 0.81).

In summary, it can be seen that both in the casuistic observations and in the high volume single and multicenter reports, both in the group of isolated PM and in the group that added to isolated PM also individual observations of PM with resectable extrapancreatic metastases, there was no influence of single or multiple occurrence of PM on the SRs.

#### 3.2.2. Number of Pancreatic Metastases

From 336 analysed casuistic observations, no relationship could be found between the number of PM and SR (HR 0.916; 95% IV 0.730–1.135; *p* = 0.419). The only major institutional report that examined this question (in a group that added to isolated PM also individual observations of PM with resectable extrapancreatic metastases) also showed no influence of the PM number on the SR: Di Franco et al. [232], N = 21, n. s. (*p* = 0.823).

#### 3.2.3. Size of PM

Adequate information was available for 253 casuistic communications. Analysis showed that there was no relationship between metastasis size and survival: HR 1. 014, 95% IV 0. 999–1. 028; *p* = 0.188. Large single and multicentre reports show the consistent picture of a missing influence of size on survival in isolated PM: Konstantinidis et al. [143] N = 20, n. s. (*p =* 0.78); Tosoian et al. [183] N = 42, n. s. (*p* = 0.602); Reddy et al. [259], n. s. (*p* = 0.17). This result is further confirmed in a 10-year literature review that also found no relationship between metastases size and SR (Dong et al. [194] N = 95, n. s. (*p* = 0.87). In publications that also included individual PM with simultaneously resected extrapancreatic metastases in their collectives, there are consistent results: Schwarz et al. [181] N = 62, n. s. (*p* = 0.93); Di Franco et al. [232] N = 21, n. s. (*p* = 0.569); Milanetto et al. [235] N = 39, n. s. (*p* = 0.80); Blanco-Fernándes et al. [243] N = 116, n. s. (*p* = 0.81). In summary, even for the parameter “size of metastases”, the communications presented do not show any significant impact on the OCTs. 

### 3.3. Risk Factors Related to the Time of PM Operation

#### 3.3.1. Synchronous Versus Metachronous Occurrence

**Figure 4:**
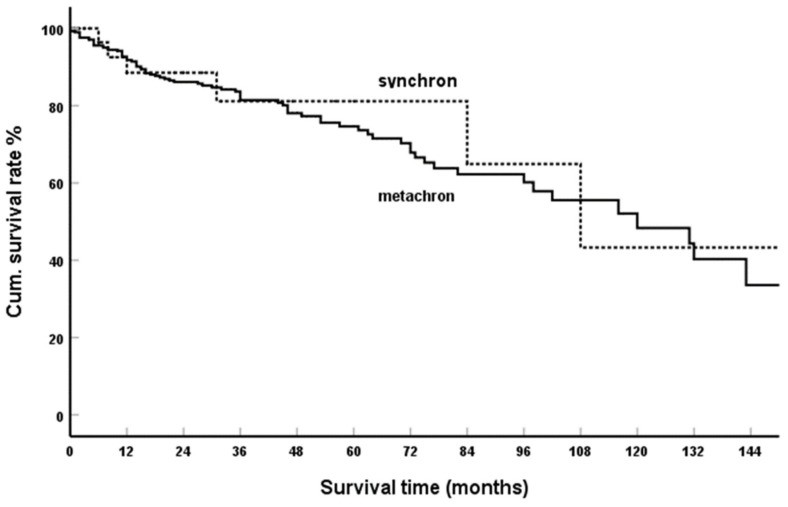
Synchronous vs. metachronous isPMRCC; Kaplan-Meier survival curves (*p* = 0.757).

Detailed information on these factors was provided in 453 casuistic communications. The result of the meta-analysis is shown in Figure 4 with a cumulative five-year SR of 64.9% and 75.7%, respectively. The difference is not statistically significant (*p* = 0.757). Large single and multicentre reports on isPMRCC also found no dependence of survival time on synchronous or metachronous metastases: Tosoian et al. [183] N = 42, n. s. (*p* = 0.509); Malleo et al. [245] N = 69, n. s. (*p* = 0.55); Reddy et al. [259] N = 21, n. s. (*p* = 0.98).

In the publications that included cases of PM with synchronously resected extrapancreatic metastases as well, the following results were found in the literature: Milanetto et al. [235] N = 29, *p* < 0. 001; Blanco-Fernandes et al. [243] N = 116, n. s. (*p* = 0.45). Results from literature compilations are similar: Dong et al. [194] N = 199, n. s. (*p* = 0.91); Masetti et al. [144] N = 187, n. s. (*p* = 0.092); Tanis et al. [137] N = 293, n. s. (*p* = 0.509). Thus, in summary, the results in isolated PM, both the casuistic and the single and multicenter reports, provide the consistent result of a lack of influence of synchronous vs. metachronous progression on survival. This uniform picture of isolated PM is contrasted with the result when observations with extrapancreatic metastases are included in the analyses, while one author also documented a lack of influence [243], the other saw a dependence of SR on synchronous or metachronous occurrence [235].

#### 3.3.2. Interval between Nephrectomy and PM Diagnosis

No relevance between interval and survival can be documented from 420 sufficiently documented case reports: HR 1.004; 95% IV 0971–1.038; *p* = 0.830. For isolated PM, the only major single institutional report published so far confirmed this result: Tosoian et al. [183] N = 42, n. s. (*p* = 0.738) as well as a 10-year literature review: Dong et al. [194], N = 199, n. s. (*p* = 0.53). Reports that include both isolated PM and PM with synchronously resected extrapancreatic metastases are available in at least four large single and multicentre reports: Schwarz et al. [181] N = 62, n. s. (*p* = 0.73); Di Franco et al. [232] N = 21, n. s. (*p* = 0.143); Milanetto et al. [235] N = 33, n. s. (*p* = 0.96); Blanco-Fernándes et al. [243] N = 116, *p* = 0.03. In summary, the studies on the influence of the interval show that as long as only isolated PM are considered, there is no evidence of a dependence between interval and OS. Only when PM observations with extrapancreatic metastass are included does this homogeneous picture change; in at least one of four studies a significant influence occurs now [243].

### 3.4. Extent of Resection

**Figure 5:**
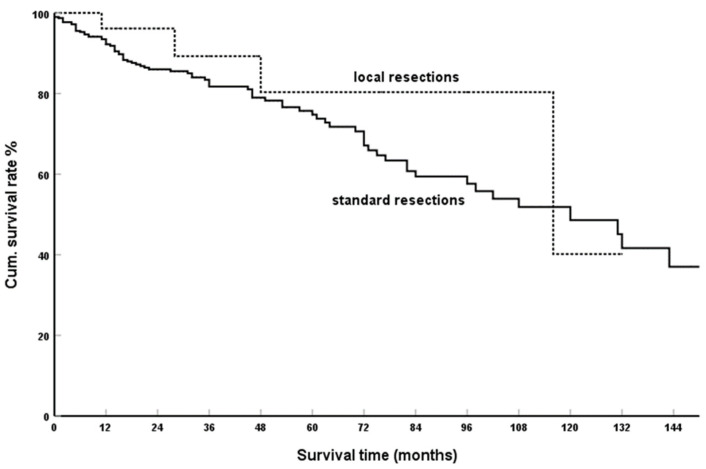
Standard vs. local resections in isPMRCC; Kaplan-Meier survival curves (*p* = 0.252).

The comparison between standard resections (N = 394) and local resections (N = 36) is shown in Figure 5. The cumulative five-year SR was 74.8% vs. 80.4% (*p* = 0.252). In the only reported high volume publication of isolated PM analysing these data, the lack of effect of extend of resection is confirmed: Malleo et al. [245] N = 69, n. s. (*p* = 0.61), as well as in a literature review by Dong et al. [194] N = 151, n. s. (*p* = 0.94). The same is true for the only work in this field, which includes cases with synchronously resected extrapancreatic metastases: Milanetto et al. [235] N = 36, n. s. (*p* = 0.43).

## 4. Discussion

### 4.1. Genetics/Epigenetics of isPMRCC

Both the spontaneous course and even more pronounced results after curative procedures with isPMRCC show unusually favourable results (spontaneous course: 3-years SR 55%; surgical therapy 5- and 10-year SR 74.8% and 48.4%, respectively). The isPMRCC thus unquestionably belongs to the disease course occurring in 20% of the mRCC cases with low tumour cell aggressiveness and protracted clinical course [131,152,174,180,193,260]. However, not only isolated PM of the RCC is characterized by a protracted course with a favourable prognosis. Additionally, in multiple organ site metastases of the RCC, the presence of PM signals a more favourable course, as Grassi et al. [261] pointed out for the first time and as confirmed by several studies [238,262,263,264,265,266,267],. Whether the favourable results in isolated PM and in PM in the course of generalization stages are based on the same pathomechanism in different degrees of severity is currently unclear. It is at least conceivable that, in the isPMRCC cases, a second mechanism is added, which triggers SSM and the particularly favourable course (see 4.3). 

Several genetic/epigenetic causes that are effective in the occurrence of PM in RCC have been at least partially deciphered in recent years. Already in 2013, the genome of the clear cell RCC was identified [268], which is characterized by the absence or mutation of the VHL tumour suppressor gene (localized at 3p25) and frequent inactivation of the chromatin-modifying genes PBRM1, BAP1 and SETD2 [269,270]. Whereas the occurrence of PAB1 mutations is associated with a poorer prognosis, BPRM1 mutations seem to be associated with a more favourable course [271]. Additionally, according to Voss et al. [272], PAB1 mutations are associated with a reduced prognosis (*p* = 0.0008), as is the absence of BPRM1 alterations (*p* = 0.0035). Turajlic et al. [273] investigated the genetic changes that control the metastasis potential of RCC and found three characteristic changes: 1. The loss of 9p21.3 and less markedly of 14q31.2 are specific changes at the beginning of the metastatic process; 2. the metastasis potential of RCC is reduced by low intratumoural heterogeneity and small proportion of somatic copy-number alterations; and 3. distinct patterns of metastasis are caused by punctual branches evolution. The authors also present three isPMRCC observations in detail that show a genetic profile different from RCC metastases in other organs: The lack of 9p loss and a significant lower weight genome instability index, which underlines a high genetic stability of these tumour cell clones. Singla et al. [238] finally uncovered, in 2020, the genetic characteristics of PM of the RCC, which occurred either isolated or as part of a multi-organ site metastasing RCC. In PM cell clones, they found changes that were associated with less aggressiveness of the disease, such as low rate of PAB1 (3%) and high rate of BPRM1 alterations (77%). Furthermore, they also found evidence of a relative genetic stability of the tumour cells, as they report that limited diversification is observed in the primary tumours leading to PM but also in the PM themselves and summarize that tumours and metastases from patients with PM are characterized by a limited spectrum of alterations consistent with a constrained evolutionary process. Thus, the occurrence of PM in RCC is linked to genetic changes that are associated with low aggressiveness of the cell clones, such as lack of 9p and 14q loss, low weight genome instability index, low frequency of BAP1 alterations and high frequency of BPRM1 loss.

### 4.2. IsPMRCC and Risk Factors

Early literature reports advised against radical operations in synchronous or multiple isPMRCC, since a poor prognosis was feared in these cases [81,90,93,161,174]. However, this assumption had to be discarded already with the first major literature collections [106,137] and high volume (N > 20) institutional reports [181,183,184,259], since it was not possible to prove that the OS was dependent on the synchronous or multiple occurrence of PM. This implies that patients with multiple or synchronous PM are as good candidates for surgical therapy as patients with singular or very late PM. Since then, numerous studies have shown that not only synchronous vs. metachronous [137,183,194,235,243,245,259] and single vs. multiple [137,143,181,183,184,194,215,232,235,243,245,259], but also size [143,181,183,194,232,235,243,259] and number [232] of PM, the interval to the occurrence of PM [181,183,194,232,235,243] and the extent of resection: Local vs. standard resections [194,235,245] do not significantly influence the prognosis. In addition to the name-giving peculiarity of isolated PM, there is the additional clinical peculiarity that these risk factors are not effective in isPMRCC. This behaviour fully affects the observations with isolated PM of the RCC, i.e., cases in which metastases occur exclusively in the pancreas. No influence of these risk factors could be identified in all such literature releases. The inclusion of individual observations of PM with synchronously resected extrapancreatic metastases in individual studies may weaken this behaviour; synchronous occurrence [235] and interval [243] was identified as a negative risk factor in at least two such publications. Therefore, according to the results presented here, it cannot be excluded that the latter group is associated with a more unfavourable outcome, even if two individual studies did not observe a significant difference between the two groups [195,229]. 

Considering isPMRCC alone, however, apart from the specificity of isolated pancreatic infestation, a further specificity is the independence of the prognosis from risk factors, whose validity is well documented for other oncological indications. After metastasectomy of pulmonary RCC metastases, the foremost site of RCC metastases, number and size of the lesions and interval to the occurrence of metastases have been identified in a variety of publications as predictors of outcome [274,275,276,277,278]. The same applies to liver metastases in RCC. Additionally, the size and number of metastases, metachronous occurrence and interval were identified as risk factors [279,280,281,282]. This also applies to the metastases of other primary tumours. These risk factors are equally effective in colorectal cancer metastasis surgery (liver metastases [283,284,285,286,287,288,289]; pulmonary metastases [290,291,292,293]). Thus, after clinical R0 resection of organ metastases, a large number of studies show that both the overall tumour burden (singular/multiple metastases, size or number of metastases) and growth rate dependent factors (synchronous/metachronous metastases, interval to onset) influence the outcome. It is therefore all the more striking that the isPMRCC entity behaves to the contrary, which needs to be explained. The same applies to the lack of influence of the extent of surgical therapy. The importance of local resection procedures in the therapy of isPMRCC is controversially discussed in the literature. In addition to the effects of the minor surgical trauma (e.g., on immunosurveillance), the lower pancreatic parenchymal loss is mentioned by the proponents [64,135,142,294]. The opponents, in turn, warn against the increased risk of recurrence and the imminent risk of overlooked regional LN metastases and the resulting poorer prognosis [99,181]. The result obtained from the presented meta-analysis, that there is no difference in survival between local and standard resections (see 3.4), is therefore unexpected and also asks for an explanation. 

Before discussing the possible causes of this strange behaviour of isPMRCC, it is necessary to point out the principle of action of these risk factors. All of the risk factors discussed have in common that they are ultimately only an expression of the magnitude of the probability that clinically undetectable, occult micrometastases are already present outside the pancreas at the time of surgery, leading later to tumour progression. It is plausible that with increasing tumour burden, the mass of tumour cells increases as does the number of tumour vessels, and thus the risk of tumour vessel infiltration and embolized tumour cells. It is equally plausible that with an early onset of metastases, i.e., a faster tumour growth, there is an increased probability that additional occult metastases are already present at the time of surgery. However, what prevents this mechanism from having an effect on the isPMRCC? 

### 4.3. IsPMRCC and Seed and Soil Mechanism

The definite or at least for a very long time exclusive occurrence of PM in RCC cannot, as initially assumed, be due to the topographic proximity of the kidney and pancreas via pre-existing lymphatic [22,66,79,84,138,154,161,177] or venous renal-portal vascular connections between the kidney and the nearby pancreas [84,295,296] or by acquired pathological tumour vessels of hyper-vascularised tumours [10,22,66,79,84,115,138,153,161,173,205]. As our working group pointed out already in 2018, the local metastasis pathway is not compatible with the following characteristics of the isPMRCC [297]: (1) The independence of the distribution of metastases in the pancreas from the side of the primary RCC [106,143,160,183,184,188,211,233,259,297,298], (2) the even distribution of isolated PM within the pancreas [299], (3) the rare occurrence (6.2% [300]) of regional LNN metastases [99,102,111,116,121,143,162,176,179,181,183,184,186,188,209,211,222,230,232,235,243,251], (4) the observation that of the few cases of extrapancreatic metastases diagnosed and removed between RCC surgery and the onset of PM, 78.3% were unquestionably of systemic haematogenic origin [22,55,62,63,66,81,99,124,131,134,145,155,163,169,187,188,193,205,228,230], (5) with a value of 8% [299] the rate of subsequent liver metastases is 2022 (24/288) not increased [10,64,68,79,80,84,106,118,134,135,154,155,169,180,217,230], and (6) recently reported for the first time the simultaneous occurrence of multiple isolated pancreatic metastases and a metastasis in an ectopic pancreas [254]. These are factors that can only be reconciled with a high importance of a systemic hematogenic metastatic pathway, since only this pathway can produce an even distribution of metastases in the pancreas (points 1, 2, 4 and 6). (Conversely the local tumour cell spread should offer for right sided RCC a greater chance to metastasize in the near caput pancreatis and for left sided RCC in the nearby corpus and cauda pancreatis, thus resulting in a dependence of metastasis localisation from RCC side. However, this is not the case, which confirms a low importance of the local metastatic pathway. The low rate of LN and liver metastases (points 3 and 5) also allows only a minor significance of the local lymphatic and local venous, i. e. direct renal-portal metastatic pathway, since this would have to be associated with an increased rate of LNN and liver metastases). However, if epidemiological studies suggest that systemic hematogenic metastasis is highly valued in the isPMRCC, this inevitably leads to the next question: Why, despite the systemic hematogenic metastasis pathway, do the metastases occur only in the pancreas? According to current knowledge, this can only be explained by the assumption of a simultaneous selection mechanism—a SSM that allows embolized RCC cells to settle and grow to metastases only in the pancreas [297]. The SSM discovered in 1889 by Paget [301] means that the pattern of metastatic spread of cancer is not random, but that the individual tumour entities are assigned preferred host organs [302]. He aptly called this behaviour “Seed and Soil Mechanism”, since successful metastasis formation is the result of a multi-stage, cascade-shaped interaction of cancer cell properties (seed) with those of the host organ (soil). The settlement and growth of an embolized tumour cell to clinical metastasis can and will therefore only take place in an organ in which the respective properties of the host and the tumour cell exactly match each other. Even blocking a single step in this complex process can make metastasis impossible [303,304,305,306]. The hypothesis of the existence of an exquisite SSM in isPMRCC [297] is now able to explain all the peculiarities of this entity without compulsion, if the SSM is so exquisitely pronounced that, (a) only the formation of PM is permitted, while (b) all other extrapancreatic embolised renal cancer cells are either definitively destroyed or “arrested” for years and decades in a dormant state [307,308]. The lack of vital extrapancreatic tumour cell nests capable of metastasis formation can now, in addition to the isolated occurrence of PM and the good surgical treatment results, also explain the unconstrained lack of significance of those risk factors, which only reflect the probability of the presence of occult extrapancreatic micrometastases. Since the highly specific and highly effective SSM in isPMRCC causes this probability to tend towards zero in extrapancreatic organs, there are no viable extrapancreatic tumour cell nests and these risk factors must remain ineffective. The lack of efficacy of the mentioned risk factors is therefore not a second independent characteristic of the tumour cells in isPMRCC, but an inevitable consequence of the postulated SSM. The same applies to the interpretation of the non-different treatment results of local and standard resections. As possible explanation for the non-different treatment results of local and standard resections, the hypothesis of the above mentioned SSM must be pointed out again. Since this causes the extrapancreatic absence of viable tumour cells or cells capable of metastasis formation, extensions of the resection boundaries will not lead to an improvement in the results. However, the SSM cannot be solely responsible for the protracted course. It is equally essential for the favourable course that these tumour cell properties remain constant over a period of years and decades. This behaviour has been confirmed by genetic studies which have shown that the occurrence of PM in RCC is associated with cell clones with relatively increased genetic stability [238,273]. So, according to these data, the step-by-step dedifferentiation of the tumour cells with increasing tumour age is unusually low in isPMRCC. This certainly differs from the behaviour more common in malignant tumours, that as the tumour continues to develop (be it primum or metastasis), more and more undifferentiated and aggressive cell clones emerge and prevail, which ultimately determine the fatal clinical course.

### 4.4. IsPMRCC and Organotropism

The exact cause of this highly specific SSM is (yet) unexplored due to the rarity of the isPMRCC. At present, therefore, only those mechanisms that can trigger organotropism and that have been found in more frequent and better studied tumours can be presented and discussed [299,309,310,311]. (1) The premetastatic niche (pmN) [302,312,313]. This results from the ability of tumours to “manipulate” a peripheral host organ even before the appearance of metastases in such a way that a special microenvironment is created in it, which enables the formation of metastases by inflammation, immunosuppression, increased angiogenesis, vascular leakiness and extracellular matrix remodelling [306,314,315,316]. Since the formation of pmN results from an interaction of primary tumour cell derived components (exosomes, microvesicles) [311,312,317,318,319,320], tumour mobilised bone marrow derived cells (e.g., MDSC, TAM) [312,319,321] and the local microenvironment [319,322] this is associated with organotropism. (2) The chemokine receptor–ligand mechanism is a necessary prerequisite for the activation of numerous signal transforming pathways, which are critical in the early metastatic process [323,324]. The chemokine receptors transduce intracellular signals by binding with their homologous ligands, which is crucial for premetastatic recruitment of specific cells e. g. regulatory T cells and tumour colonization [313,324]. Since the chemokine receptor is specific to the tumour cell and the ligand to a host organ, a successful interaction can only take place in organs where the receptor and ligand match exactly, which again triggers an organotropism. This mechanism could be blamed early on for the metastasis behaviour of breast cancer [303]. (3) The metabolic adaptation [310,325]. It is essential for the survival of tumour cells in early avascular metastasis growth, as the supply with energy carriers by diffusion alone is critical. Therefore, those cell clones will gain an advantage in metastasis formation that are able to make the best use of locally available energy sources [310,325,326,327,328,329,330], by overcoming metabolic barriers by metabolic plasticity, which enables them to use all resources available in an individual organ. Since tumour and host cell properties also have to match in this mechanism, this also leads to organotropism in the development of metastases. (4) Immune-surveillance. An intact immune system is able to recognise tumour cells and combat them as foreign cells. The importance of the immune system in RCC was assumed early on by the extremely rarely observed spontaneous remission of metastases, also in the pancreas [63], which were attributed to changes in the immune defence [331,332,333]. A dangerous counter-strategy of malignant tumours is therefore their ability to block the immune response. This realization led to the introduction of IT [258,334,335,336], which tries to restore the immune system and which also proved to be effective in RCC. The result of a study by Singla [238] was therefore surprising: in PM of mRCC, immunotherapy (nivolumab) was ineffective, whereas treatment with angiogenesis inhibitors was highly effective. This observation was matched by the behaviour of the biomarkers determined: while angiogenetic markers were increased, inflammatory markers remained low. This suggests that the occurrence of PM in mRCC is linked to the non-inflammatory subtype [337]. This is characterized by enrichment of endothel cells, low frequency of macrophages, B cells, T cells, NK cells, and neutrophils, marked BPRM1 gene loss, and increased angiogenesis, and thus with a good response to TKI therapy. On the other hand, the lack of inflammatory components explains a non-response to IT [238,338]. This suggests that the tumour cells in this rare tumour entity are recognised and fought by the body’s own immune system. Why the immune defence is ineffective in the pancreas alone and leads to organotropism remains unclear. 

Limitations of the analysis are its retrospective character and the very long period of investigation (1952–2022). The possibility of bias applies first to casuistic reports in which the indication for surgery is influenced by the individual surgeon and the time of surgery, and for which the chance of publication is greater for unusual courses, be they particularly good, bad or rare, than for “standard” observations. In the case of the large single and multicentre reports, the non-randomized study design, which is retrospective and covers very long periods of time, must also be regarded as a limitation [298], which can cause bias in the case selection. However, these methodological limitations do not seem to have a major impact overall, as the various casuistic, single and multicentre reports produced similar results.

## 5. Conclusions

In addition to the peculiarity of the exclusive metastasis growth in the pancreas other equal unusual peculiarities of isPMRCC are the ineffectiveness of risk factors associated with the overall tumour burden, the rate of growth and the extend of surgery. All these facts can be explained by the hypothesis of a very exquisite SSM, that allows metastasis growth only in the pancreas, while all extrapancreatic embolised tumour cells are either eliminated or locked into a dormant state. The absence of growth-capable extrapancreatic tumour cells has three effects: (1) Resection of the PM leads to exquisite treatment results, (2) it explains the independence of the results from the extent of resection (if an R0 situation has been achieved) and (3) it inevitably results in the ineffectiveness of the abovementioned risk factors. 

## References

[1] Jenssen E. (1952). A metastatic hypernephroma to the pancreas. Acta Chir. Scand..

[2] Lawson L., Holt L., Rooke H. (1966). Recurrent duodenal haemorrhage from renal carcinoma. Brit. J. Urol..

[3] Franciosi R.A., Russo J. (1969). Renal cell carcinoma metastatic to the pancreas thirteen years following nephrectomy. Mil. Med..

[4] Marquand J., Giraud B., Maliakas S. (1971). Pancreatic metastasis revealing a kidney neoplasm. J. Urol. Nephrol..

[5] Guttman F., Ross M., Lachance C. (1972). Pancreatic metastasis of renal cell carcinoma treated by total pancreatectomy. Arch Surg..

[6] Gillet M., Camelit G., Runser G., Clement D. (1974). Duodenopancreatic metastasis of kidney cancer revealed by digestive hemorrhage treated by cephalic duodeno-pancreatectomy. Chirurgie.

[7] Hermanutz K.D., Sonnenberg G.E. (1977). Late metastasis of a hypernephroid kidney carcinoma to the pancreas with tumor invasion to the duodenum. Fortschr. Röntgenstr..

[8] Saxon A., Gottesman J., Doolas A. (1980). Bilateral hypernephroma with solitary pancreatic metastasis. J. Surg. Oncol..

[9] Yazaki T., Ishikawa S., Ogawa Y., Takahashi S., Nemoto S., Rinsho K., Kanoh S., Kitagawa R. (1981). Silent pancreatic metastasis from renal cell carcinoma diagnosed at arteriography. Acta Urol. Jpn..

[10] Py J.M., Arnaud J.P., Cinqualbre J., Adloff M., Bollack C. (1984). Pancreatic metastases of nephro-epitheliomas. Apropos of 2 cases. Acta Chir. Belg..

[11] Skaarup P., Jorgensen T., Larsen S. (1984). Asynchronous metastasizing renal cell carcinoma associated with progressive immune complex glomerulonephritis and proteinuria. Scand. J. Urol. Nephrol..

[12] Audisio R.A., La Monica G. (1985). Solitary pancreatic metastasis occurring 20 years after nephrectomy for carcinoma of the kidney. Tumori.

[13] Kishimoto H., Niumra Y., Okamoto K., Tsuchie K., Yamase H., Maeda S., Kamija J., Hasagawa H., Hayakawa N., Yamamoto M. (1985). A case of resected renal cell carcinoma with massive pancreatic metastasis. Jap. J. Cancer. Clin..

[14] Amamiya H., Iizumi T., Yazaki T., Waku M., Yasuda H., Takada T., Shikata J., Nagai J. (1988). A solitary pancreatic metastasis from renal cell carcinoma. Hinyouki Geka.

[15] Carini M., Selli C., Barbanti G., Bianchi S., Muraro G. (1988). Pancreatic late recurrence of bilateral renal cell carcinoma after conservative surgery. Eur. Urol..

[16] Hirano M., Douden K., Bantou H., Sakatoku M., Saitoh H., Tachikawa H., Kitagawa K., Yamahata T., Hirano A., Kawaguchi M. (1988). Solitary pancreatic metastasis occuring 10 years after nephrectomy for carcinoma of the kidney. Tan Sui.

[17] Sharma S.K., Kumar A., Madhusoodnan P., Banerjee C., Suri S., Dhar M. (1988). Solitary pancreatic metastasis from renal cell carcinoma. A rare metastatic site. Indian J. Cancer.

[18] Guyenne C., Rat P., Haas O., Baudet J.G., Favre J.P. (1989). Triple metastase pancreatique d´un cancer du rein traitee par duodenopancreatectomie subtotale. Presse Med..

[19] Iwanami M., Nakayoshi A., Yagi H., Shimizu K., Kimura K., Suzuki K., Matsumoto K., Kai Y., Heno M., Sagawa F. (1989). A resected case of the asymptomatic pancreatic metastasis in the body and tail of the pancreas from renal cell carcinoma. J. Jpn. Panc. Soc..

[20] Roland C., van Heerden J. (1989). Nonpancreatic primary tumors with metastasis to the pancreas. Surg. Gynecol. Obstet..

[21] Simpson N.S., Mulholland C.K., Lioe T., Spence R. (1989). Late, solitary metastatic renal carcinoma in the pancreas. Ulster Med. J..

[22] Strijk S.P. (1989). Pancreatic metastases of renal cell carcinoma: Report of two cases. Gastrointest. Radiol..

[23] Temellini F., Bavosi M., Lamarra M., Quagliarini P., Giuliani F. (1989). Pancreatic metastasis 25 years after nephrectomy for renal cancer. Tumori.

[24] Gohji K., Matsumoto O., Kamidono S. (1990). Solitary pancreatic metastasis from renal cell carcinoma. Hinyokika Kiyo.

[25] Terashima M., Abe H., Suga K., Matsuya F., Kobayashi K., Itoh S., Sasaki R., Kanno S., Saito K., Tomichi N. (1990). Two cases of renal cell carcinoma metastasized to the pancreas and to the gallbladder. Jpn. J. Gastroenterol. Surg..

[26] Furukawa T., Hattori R., Ohtake H., Souma T., Kinukawa T., Hirai K., Kimura J., Sakata T., Ishii M., Hayashi N. (1991). A resectable case of pancreatic head metastasis from renal cell carcinoma. Hinyouki Geka.

[27] Kubo K., Morita J., Mizoe J., Ogawa H., Irie G. (1991). Renal cell carcinoma metastatic to the pancreas 8 years following nephrectomy. Jpn. J. Clin. Radiol..

[28] Nishida O., Matsunaga Y., Dekigai H., Um S., Hsieh C., Kimura F. (1991). Three elderly cases of renal cell carcinoma with pancreatic metastasis. Nippon Ronen Igakkai Zasshi.

[29] Oka H., Hatayama T., Taki Y., Ueyama H., Hida S., Noguchi M. (1991). A resected case of renal cell carcinoma with metastasis to the pancreas. Hinyokika Kiyo..

[30] Tabata T., Kuroda Y., Nishimatsu S., Satoh Y. (1991). A resected case of pancreatic tumor metastasized from renal cell carcinoma. J. Jpn. Panc. Soc..

[31] Yamamoto S., Tobinaga K., Taketomi K., Kimino K., Ashizuka S., Kishikawa M. (1991). Pancreatic metastasis of renal cell carcinoma occurring 17 years after nephrectomy. J. Jpn. Soc. Clin. Surg..

[32] Fujii M., Kogawa T., Matsuyama K., Yamamoto H., Kaawahito Y., Iinuma S., Kokura S., Takemura S., Yoshikawa T., Kondo M. (1992). A case of metastatic renal cell carcinoma to pancreas ten years after nephrectomy. J. Kyoto Pref. Univ. Med..

[33] Melo C.R., Melo I.S., Monteiro A.Z., de Mello E.S. (1992). Pancreatic metastasis from renal cell carcinoma. Arq. Gastroenterol..

[34] Nakagawa K., Tsuchiya T., Momono S., Sasaki Y., Sato T. (1992). A case of pancreatic metastasis of renal cell carcinoma. Jpn. J. Gastroenterol. Surg..

[35] Rypens F., Van Gansbeke V., Lambilliotte J., Regemorter V., Verhest A., Struyven J. (1992). Pancreatic metastasis from renal cell carcinoma. Br. J. Radiol..

[36] Stankard C., Karl R.C. (1992). The treatment of isolated pancreatic metastases from renal cell carcinoma: A surgical review. Am. J. Gastroenterol..

[37] Aikou S., Tokura Y., Yamafuji K., Takahashi T., Yoshibide O., Kishii K., Fujii S., Katsumata K., Tamiya M., Takahashi T. (1993). A resected case of pancreatic metastasis from renal cell carcinoma presenting with acute duodenal bleeding. J. Jpn. Soc. Clin. Surg..

[38] Calmes J.M., Meyer A. (1993). Pancreatic hypernephroma manifested by a duodenal hemorrhage. Rev. Med. Suisse Rom..

[39] Ishikawa T., Horimi T., Majima K. (1993). A resected case of pancreatic tumor metastasized from renal cell carcinoma. A review of 11 cases in the japanese and 13 cases in the foreign literature. J. Jpn. Soc. Clin. Surg..

[40] Kawaguchi T., Tsunoda T., Tanaka Y., Saika Y., Ohiani H., Fujii R., Zaitsu Y., Tanimura H., Takifuji K., Saika Y. (1993). A case of resection of a solitary pancreatic metastasis of renal cell carcinoma occuring 5 years after nephrectomy. J. Jpn. Panc. Soc..

[41] Marcote-Valdivieso E., Arlandis F., Baltasar A., Martinez C., Vierna G. (1993). Synchronous pancreatic metastasis of renal carcinoma. Rev. Esp. Enferm. Dig..

[42] Nan Y., Kuno N., Kurimoto K., Nakamura T., Kobayashi S. (1993). A resected case of pancreatic tumor metastasized from renal cell carcinoma diagnosed by endoscopic biopsy through the main pancreatic duct. Gastroenterol. Endosc..

[43] Oda K., Itoh J., Hachisuka K., Yamaguchi A., Isogai M., Utsunomiya H., Osamura Y., Watanabe K. (1993). Value of computer image analysis in improving ERCP images in metastatic tumor of the pancreas. AJR.

[44] Reale D., Squillaci S., Guarino M., Milesi F., Forloni B., Vezzini V., Pascale M., Micoli G., Milesi F., Forloni B. (1993). Late pancreatic metastasis of renal carcinoma. Description of 2 cases and review of literature. Minerva Urol. Nefrol..

[45] Sauvanet A., Barthes T., Levy P., Flejou J.F. (1993). Late pancreatic metastasis from renal cell carcinoma. Pancreas.

[46] Takeuchi H., Konaga E., Harano M., Watanabe K., Takeuchi Y., Hara M., Mano S. (1993). Solitary pancreatic metastasis from renal cell carcinoma. Acta Med. Okayama.

[47] Vergara V., Marucci M., Marcarino C., Brunello F., Capussotti L. (1993). Metastatic involvement of the pancreas from renal cell carcinoma treated by surgery. Ital. J. Gastroenterol..

[48] Yanagisawa T., Nakayama K., Kashiwagi M., Tanaka J., Kashiwagi T., Mizusaki K., Itoh A., Akimoto H., Takahashi T., Aoki T. (1993). Three cases of resectable pancreatic metastases from renal cell carcinoma. Geka Shinryo.

[49] Zugel N., Leipprand F., Weckermann D., Witte J. (1994). Solitäre Pankreaskopfmetastase bei hypernephroidem Carcinom. Fortschr. Med..

[50] Dousset B., Andant C., Guimbaud R., Roseau G., Tulliez M., Gaudric M., Palazzo L. (1995). Late pancreatic metastasis from renal cell carcinoma diagnosed by endoscopic ultrasonography. Surgery.

[51] Fabre J., Rounanet P., Dagues F., Blanc F., Baumel H., Domergue J. (1995). Various features and surgical approach of solitary pancreatic metastasis from renal cell carcinoma. Eur. J. Surg. Oncol..

[52] Onishi T., Ohishi Y., Iizuka N., Suzuki Y., Shirakawa H., Hatano T., Tomita M. (1995). Clinical characteristics of 7 renal cell carcinoma patients developing a solitary pancreatic metastasis after nephrectomy. Nippon Hinyokika Gakkai Zasshi.

[53] Orita M., Morita N., Hiraoka H., Noshima S., Takaimashi T., Esato K. (1995). A case of resected pancreatic metastasis from renal cell carcinoma 14 years after radical nephrectomy. J. Jpn. Panc. Soc..

[54] Takashi M., Takagi Y., Sakata T., Shimoji T., Miyake K. (1995). Surgical treatment of renal cell carcinoma metastases: Prognostic significance. Int. Urol. Nephrol..

[55] Barras J.P., Baer H., Stenzl A., Czerniak A. (1996). Isolated late metastasis of a renal cell cancer treated by radical distal pancreatectomy. HPB Surg..

[56] Hirota T., Tomida T., Iwasa M., Takahashi K., Kaneda M., Tamaki H. (1996). Solitary pancreatic metastasis occurring eight years after nephrectomy for renal cell carcinoma. A case report and surgical review. Int. J. Pancreatol..

[57] Palazzo L., Borotto E., Cellier C., Roseau G., Chaussade S., Couturier D., Paolaggi J. (1996). Endosonographic features of pancreatic metastases. Gastrointest. Endosc..

[58] Paz A., Koren R., Gal R., Wolloch Y. (1996). Late solitary pancreatic metastasis from renal cell carcinoma. Isr. J. Med. Sci..

[59] Chambers T., Fishman E., Hruban R. (1997). Pancreatic metastases from renal cell carcinoma in von Hippel-Lindau disease. Clin. Imaging.

[60] Harrison L.E., Merchant N., Cohen A.M., Brennan M.F. (1997). Pancreaticoduodenectomy for nonperiampullary primary tumors. Am. J. Surg..

[61] Robbins E.G., Franceschi D., Barkin J. (1997). Solitary metastatic tumors to the pancreas: A case report and review of the literature. Am. J. Gastroenterol..

[62] Adem C., Chetritt J., Guymar S., Bellil K., Ladouch-Badre A., Benlagha N., Bedossa P. (1998). Pancreatic metastasis of a renal adenocarcinoma. Apropos on 2 cases. Ann. Pathol..

[63] Altschuler E., Ray A. (1998). Spontaneous regression of a pancreatic metastasis of a renal cell carcinoma. Arch. Fam. Med..

[64] Butturini G., Bassi C., Falconi M., Salvia R., Caldiron E., Iannucci A., Zamboni G., Grazinai R., Procacci C., Pederzoli P. (1998). Surgical treatment of pancreatic metastases from renal cell carcinomas. Dig. Surg..

[65] Gupta R.K., Lallu S., Delahunt B. (1998). Fine-needle aspiration cytology of metastatic clear-cell renal carcinoma presenting as a solitary mass in the head of the pancreas. Diagn. Cytopathol..

[66] Hashimoto M., Watanabe G., Matsuda M., Dohi T., Tsurumaru M. (1998). Management of pancreatic metastases from renal cell carcinoma: Report of four resected cases. Hepatogastroenterology.

[67] Jingu K., Watanabe K., Yamamoto H., Fujita Y., Honda I., Watanabe S., Nagata M., Sugimoto K., Watanabe Y., Fujita Y. (1998). Surgical treatment of a solitary pancreatic metastasis from renal cell carcinoma: Report of a case. Surg. Today.

[68] Merkle E.M., Boaz T., Kolokythas O., Haaga J.R., Lewin J.S., Brambs H.J. (1998). Metastases to the pancreas. Br. J. Radiol..

[69] Sahin M., Foulis A.A., Poon F.W., Imrie C.W. (1998). Late focal pancreatic metastasis of renal cell carcinoma. Dig. Surg..

[70] Z´graggen K., Fernandez-del Castillo C., Rattner D., Sigala H., Warshaw A. (1998). Metastases to the pancreas and their surgical extirpation. Arch. Surg..

[71] Augustin H., Bacher H., Uggowitzer M., Ott A., Hubmer G., Mischinger H. (1999). Pancreatic metastases from renal cell carcinoma mimicking insulinomas. BJU Int..

[72] Carucci L., Siegelman E., Feldman M. (1999). Pancreatic metastasis from clear cell renal carcinoma: Diagnosis with chemical shift MRI. J. Comput. Assist. Tomogr..

[73] Eriguchi N., Aoyagi S., Hara M., Miyazaki T., Hashino K., Imamura I., Jimi A., Naito H., Miyazaki T., Hashimo K. (1999). A resected case of pancreatic metastasis from primary renal cell carcinoma. Kurume Med. J..

[74] Ng C.S., Loyer E.M., Iyer R.B., David C.L., DuBrow R.A., Charnsangavej C. (1999). Metastases to the pancreas from renal cell carcinoma: Findings on three-phase contrast-enhanced helical CT. AJR.

[75] Sugiyama M., Katsura M., Yamamoto K., Nouchi W., Abe N., Hatano N., Atomi Y. (1999). Pancreatic metastasis from renal cell carcinoma causing massive gastrointestinal bleeding in von Hippel-Lindau disease. Hepatogastroenterology.

[76] Yavaşçaoğlu I., Korun N., Oktay B., Simsek U., Ozyurt M. (1999). Renal cell carcinoma with solitary synchronous pancreaticoduodenal and metachronous periprostatic metastases: Report of a case. Surg. Today.

[77] Fricke P., Schulz H.U., Buhtz B., Lippert H. (2000). Multiple metachrone Metastasen eines Nierenzellkarzinoms im Pankreas. Fallbeschreibung und Literaturübersicht. Chirurg.

[78] Ghavamian R., Klein K.A., Stephens D.H., Welch T.J., LeRoy A.J., Richardson R.L., Burch P.A., Zincke H. (2000). Renal cell carcinoma metastatic to the pancreas: Clinical and radiological features. Mayo Clin. Proc..

[79] Kassabian A., Stein J., Jabbour N., Parsa K., Skinner D., Parekh D., Cosenza C., Selby R. (2000). Renal cell carcinoma metastatic to the pancreas: A single institution series and review of the literature. Urology.

[80] Le Borgne J., Partensky C., Glemain P., Dupas B., de Kerviller B. (2000). Pancreaticoduodenectomy for metastatic ampullary and pancreatic tumors. Hepatogastroenterology.

[81] Mehta N., Volpe C., Haley T., Balos L., Bradley E.L., Doerr R.J. (2000). Pancreaticoduodenectomy for metastatic renal cell carcinoma: Report of a case. Surg. Today.

[82] Thompson L.D., Heffess C.S. (2000). Renal cell carcinoma to the pancreas in surgical pathology material. Cancer.

[83] Espinoza R., Rossi R., Rossi R., Rosenberg H. (2001). Metachronous pancreatic metastasis of a renal cell carcinoma: 3 new cases. Rev. Med. Chil..

[84] Faure J.P., Tuech J.J., Richer J.P., Pessaux P., Arnaud J.P., Carretier M. (2001). Pancreatic metastasis of renal cell carcinoma: Presentation, treatment and survival. J. Urol..

[85] Marusch F., Koch A., Dietrich F., Hoschke B., Gastinger I. (2001). A singular late metastasis of renal cell carcinoma inside the pancreas. An uncommon pancreatic tumor. Zentralbl. Chir..

[86] Ruibal Moldes M., Quintana de la Rosa J., Farina Perez L., Tardaguila F., Ortiz Rey J., Zungri Telo E. (2001). Late pancreatic metastasis from renal carcinoma. Actas Urol. Esp..

[87] Scatarige J., Horton K., Sheth S., Fishman E. (2001). Pancreatic parenchymal metastases: Observations on helical CT. Am. J. Roentenol..

[88] Sohn T.A., Yeo C.J., Cameron J.L., Nakeeb A., Lillemoe K.D. (2001). Renal cell carcinoma metastatic to the pancreas: Results of surgical management. J. Gastrointest. Surg..

[89] Tada T., Kobayashi G., Noda Y., Kimura K., Ito K., Fujita N. (2001). A resected case with multiple pancreatic metastasis of renal cell carcinoma. Nippon Shokakibyo Gakkai Zasshi.

[90] Béchade D., Palazzo I., Desramé J., Duvic C., Hérody M., Didelot F., Coutant G., Algayres J. (2002). Pancreatic metastasis of renal carcinoma: Report of three cases. Rev. Med. Interne.

[91] Chao K., Hurley J., Neerhut G., Kiroff G. (2002). Multiple pancreatic metastases from renal cell carcinoma. ANZ J. Surg..

[92] Chou Y., Chiou H., Hong T., Tiu C., Chiou S., Su C., Tsay S. (2002). Solitary metastasis from renal cell carcinoma presenting as diffuse pancreatic enlargement. J. Clin. Ultrasound.

[93] Eloubeidi M.A., Jhala D., Chhieng D.C., Jhala N., Eltoum I., Wilcox C.M. (2002). Multiple late asymptomatic pancreatic metastases from renal cell carcinoma: Diagnosis by endoscopic ultrasound-guided fine needle aspiration biopsy with immunocytochemical correlation. Dig. Dis. Sci..

[94] Hiotis S., Klimstra D., Conlon K., Brennan M. (2002). Results after pancreatic resection for metastatic lesions. Ann. Surg. Oncol..

[95] Lisii D., Gaimant A., Sautereau D., Paraf F., Maubon A. (2002). Duodenal bleeding revealing a renal cell carcinoma. Gastroenterol. Clin. Biol..

[96] Peschaud F., Cheynel N., Hagry O., Tremeaux J.C., Rat P., Favre J.P. (2002). Surgical treatment of pancreatic metastases from renal carcinoma. Ann. Chir..

[97] Roviello F., Nastri G., Hako L., Marrelli D., De Stefano A., Cioppa T., Pinto E. (2002). Pancreatic metastasis from clear renal cell carcinoma: A clinical case. Chir. Ital..

[98] Yachida S., Fukushima N., Kanai Y., Nimura S., Shimada K., Yamamoto J., Sakamoto M. (2002). Pancreatic metastasis from renal cell carcinoma extending into the main pancreatic duct: A case report. Jpn. J. Clin. Oncol..

[99] Bassi C., Butturini G., Falconi M., Sargenti W., Mantovavi W., Pederzoli P. (2003). High recurrence rate after atypical resection for pancreatic metastases from renal cell carcinoma. Br. J. Surg..

[100] Giulini S., Portolani N., Bonardelli S., Baiocchi G., Zampatti N., Coniglio A., Baronchelli C. (2003). Distal pancreatic resection with splenic preservation for metastasis of renal carcinoma diagnosed 24 years later from the nephrectomy. Ann. Ital. Chir..

[101] Hernandez D.J., Kavoussi L.R., Ellison L. (2003). Laparoscopic distal pancreatectomy for metastatic renal cell carcinoma. Urology.

[102] Law C.H., Wei A.C., Hanna S.S., Al-Zahrani M., Taylor B.R., Greig B., Langer B., Gallinger S., Al-Zahrani M., Taylor B. (2003). Pancreatic resection for metastatic renal cell carcinoma: Presentation, treatment and outcome. Ann. Surg. Oncol..

[103] Nakagohri T., Konishi M., Inoue K., Nakamura T., Kinoshita T. (2003). Partial pancreatic head resection for pancreatis metastasis from renal cell carcinoma. Hepatogastroenterology.

[104] Pecchi A., Cesinaro A., Torricelli P. (2003). Solitary pancreatic metastasis from renal cell carcinoma. A case report. Radiol. Med..

[105] Uemura T., Kurita A., Nishimura R., Ishizaki M., Takashima S. (2003). Solitary pancreatic metastasis from renal cell carcinoma concomitant with early gastric cancer 17 years after nephrectomy. Report of a case. Surg. Today.

[106] Sellner F., Tykalsky N., De Santis M., Pont J., Klimpfinger M. (2006). Solitary and multiple isolated metastases of clear cell renal carcinoma: An indication for pancreatic surgery. Ann. Surg. Oncol..

[107] Zacharoulis D., Asopa V., Karvounis E., Williamson R.C. (2003). Resection of renal metastases to the pancreas: A surgical challenge. HPB..

[108] Kijvikai K., Ratana-olarn K. (2004). Solitary pancreatic metastasis from renal cell carcinoma 14 years after nephrectomy: A case report. J. Med. Assoc. Thai..

[109] Kobayashi A., Yamaguchi T., Ishihara T., Tadenuma H., Nakamura K., Ohshimi T., Sakaue N., Baba T., Yoshikawa M., Saisho H. (2004). Spontaneous rupture of pancreatic metastasis from renal cell carcinoma. Jpn. J. Clin. Onco..

[110] Kornprat P., Bacher H., Hauser H., Cerwenka H., El-Shabrawi A., Lackner C., Mischinger H.J. (2004). Renal cell carcinoma with metastasis to the pancreas: A case report and literature review. Eur. Surg..

[111] Moussa A., Mitry E., Hammel P., Sauvanet A., Nassif T., Palazzo L. (2004). Pancreatic metastasis: A multicentric study of 22 patients. Gastroenterol. Clin. Biol..

[112] Paparel P., Cotton F., Voiglio E., Decaussin M., Isaac S., Caillot J.L. (2004). A case of late pancreatic metastasis from renal cell carcinoma. Prog. Urol..

[113] Ninan S., Jain P.K., Paul A., Menon K.V. (2005). Synchronous pancreatic metastases from asymptomatic renal cell carcinoma. JOP.

[114] Pekmezci S., Saribeyoglu K., Kahya A.S., Kapan M., Durgun V. (2005). Pancreatic renal cell carcinoma metastasis presenting with upper gastrointestinal bleeding. Surgery.

[115] Sotiropoulos G.C., Lang H., Liu C., Brokalaki E.I., Molmenti E., Broelsch C.E. (2005). Surgical treatment of pancreatic metastases of renal cell carcinoma. JOP.

[116] Wente M.N., Kleef J., Esposito I., Hartel M., Müller M.W., Fröhlich E., Büchler M.W., Friess H. (2005). Renal cancer cell metastasis into the pancreas: A single-center experience and overview of the literature. Pancreas.

[117] Crippa S., Angelini C., Mussi C., Bonardi C., Romano F., Sartori P., Uggeri F., Bovo G. (2006). Surgical treatment of metastatic tumors to the pancreas: A single center experience and review of the literature. World J. Surg..

[118] Köhler K., Haroske G., Ludwig K. (2006). Management of pancreatic metastases from renal cell carcinoma. Report of five cases. Zentralbl. Chir..

[119] Shrikhande S.V., Büchler P., Esposito I., Loos M., Büchler M.W., Friess H. (2006). Splenic and portal vein thrombosis in pancreatic metastasis from renal cell carcinoma. World. J. Surg. Oncol..

[120] Akatsu T., Shimazu M., Aiura K., Ito Y., Shinoda M., Kawachi S., Tanabe M., Ueda M., Kitajima M., Kitagawa Y. (2007). Clinicopathological features and surgical outcome of isolated metastasis of renal cell carcinoma. Hepatogastroenterology.

[121] Eidt S., Jergas M., Schmidt R., Siedek M. (2007). Metastasis to the pancreas—An indication for pancreatic resection?. Langenbecks Arch. Surg..

[122] Goto T., Dohmen T., Yoneyama K. (2007). Pancreatic metastasis from renal cell carcinoma. Clin. Gastroenterol. Hepatol..

[123] Karimi K.M., McFadden D.W. (2007). Pancreatic resection for metastatic renal cell carcinoma to the pancreas. Am. Surg..

[124] Maeda H., Okabayashi T., Nishimori I., Kobayashi M., Sugimoto T., Kohsaki T., Onishi S., Hanazaki K. (2007). Duodenum-preserving pancreatic head resection for pancreatic metastasis from renal cell carcinoma: A case report. Langenbecks Arch. Surg..

[125] Varker K.A., Muscarella P., Wall K., Ellison C., Bloomston M. (2007). Pancreatectomy for non-pancreatic malignancies results in improved survival after R0 resection. World. J. Surg. Oncol..

[126] Aimoto T., Uchida E., Yamahatsu K., Yoshida H., Hiroi M., Tajiri T. (2008). Surgical treatment for isolated multiple pancreatic metastases from renal cell carcinoma: Report of a case. J. Nippon Med. Sch..

[127] Bahra M., Jacob D., Langrehr J.M., Glanemann M., Schumacher G., Lopez-Hänninen E., Neuhaus P. (2008). Metastasen im Pankreas. Wann ist eine Resektion sinnvoll?. Chirurg.

[128] Kawakami H., Kuwatani M., Yamato H., Shinada K., Hirano S., Kondo S., Yonemori A., Matsuno Y., Asaka M. (2008). Pancreatic metastasis from renal cell carcinoma with intraportal tumor thrombus. Inter. Med..

[129] Matsutani T., Sasajima K., Miyamoto M., Yokoyama T., Maruyama H., Yanagi K., Matsuda A., Kashiwabara M., Suzuki S., Tajiri T. (2008). Resection of pancreatic metastasis from renal cell carcinoma and an early gastric cancer. J. Nippon Med. Sch..

[130] Koide N., Yokoyama Y., Oda K., Nishio H., Ebata T., Abe T., Igami T., Nimura Y., Nagino M. (2008). Pancreatic metastasis from renal cell carcinoma. Results of the surgical management and pathologic findings. Pancreas.

[131] Schauer M., Vogelsang H., Siewert J.R. (2008). Pancreatic resection for metastatic renal cell carcinoma: A single center experience and review of the literature. Anticancer Res..

[132] Shukla R.C., Pathak R., Senthil S. (2008). Pancreatic metastases of renal cell carcinoma—Case report. Nepal. Med. Coll. J..

[133] Tuech J., Lefebure R., Bridoux V., Albouy B., Lermite E., Le Pessot F., Le Blanc-Louvry I., Michot F. (2008). Combined resection of the pancreas and inferior vena cava for pancreatic metastasis from renal cell carcinoma. J. Gastrointest. Surg..

[134] Zerbi A., Ortolano E., Balzano G., Borri A., Beneduce A.A., Di Carlo V. (2008). Pancreatic metastasis from renal cell carcinoma: Which patients benefit from surgical resection?. Ann. Surg. Oncol..

[135] Deguchi Y., Shimada K., Nara S., Esaki M., Sakamoto Y., Kosuge T., Hiraoka N. (2009). Pancreaticojejunostomy with invagination of the punched pancreatic remnant after medial pancreatectomy and enucleation for multiple metastases of renal cell carcinoma: Report of a case. Surg. Today.

[136] Machado N.O., Chopra P. (2009). Pancreatic metastasis from renal carcinoma managed by Whipple resection. A case report and literature review of metastatic pattern, surgical management and outcome. JOP.

[137] Tanis P.J., van der Gaag N.A., Busch O.R., van Gulik T.M., Gouma D.J. (2009). Systematic review of pancreatic surgery for metastatic renal cell carcinoma. Br. J. Surg..

[138] Volk A., Kersting S., Konopke R., Dobrowolski F., Franzen S., Ockert D., Grützmann R., Saeger H.D., Bergert H. (2009). Surgical therapy of intrapancreatic metastasis from renal cell carcinoma. Pancreatology.

[139] Akashi Y., Saiura A., Kishi Y., Koga R., Morimura R., Yoshioka R., Yamamoto J., Yamaguchi T. (2010). Outcome after surgical resection of isolated metastases to the pancreas. Hepatogastroenterology.

[140] Barbaros U., Sümer A., Demirel T., Karakullukçu N., Batman B., Içscan Y., Sariçam G., Serin K., Loh W.L., Dinççag A. (2010). Single incision laparoscopic pancreas resection for pancreatic metastasis of renal cell carcinoma. JSLS..

[141] Hijioka S., Hifumi M., Mekky M., Takekuma Y., Kawaguchi T., Yokomizo H., Sato T. (2010). Total pancreatectomy for metastatic renal cell carcinoma with marked extension into the main pancreatic duct. Inter. Med..

[142] Kitasato A., Tajima Y., Kuroki T., Tsutsumi R., Tsuneoka N., Adachi T., Mishima T., Kanematsu T. (2010). Limited pancreatectomy for metastatic pancreatic tumors from renal cell carcinoma. Hepatogastroenterology.

[143] Konstantinidis I., Dursun A., Zheng H., Wargo J., Thayer S., Castillo C., Warshaw A., Ferrone C. (2010). Metastatic tumors in the pancreas in the modern era. J. Am. Coll. Surg.

[144] Masetti M., Zanini N., Martuzzi F., Fabbri C., Mastrangelo L., Landolfo G., Fornelli A., Burzi M., Vezzelli E., Jovine E. (2010). Analysis of prognostic factors in metastatic tumors of the pancreas: A single-center experience and review of the literature. Pancreas.

[145] Mourra N., Arrive L., Balladur P., Flejou J.F., Tiret E., Paye F. (2010). Isolated metastatic tumors to the pancreas. Pancreas.

[146] Szabó K.G., Szentkereszty Z., Tóth L.A., Damjanovich L., Sápy P. (2010). Distal pancreas resection for metastasis of clear cell renal cancer. Magy. Seb..

[147] Vujcic T., Brahm J., Buckel E., Ibarra A., Vial M.T., Fernández M. (2010). Pancreatic metastasis from renal cell carcinoma: A case report. Rev. Med. Chile..

[148] Yokonishi T., Ito Y., Osaka K., Komiya A., Kobayashi K., Sakai N., Noguchi S., Kishi H., Satomi Y., Mogaki M. (2010). Tsuura Y.; Mizuno N.; Ikeda I. Pancreatic metastasis from renal cell carcinoma 25 years after radical nephrectomy. Hinyokika Kiyo..

[149] D’Ambra M., Ricci C., Casadei R., Minni F. (2011). Pancreatic metastasis from renal cell carcinoma. Urologia.

[150] Irigoin R.R., Entrenas A.O., Urbano V.A., Marin J.G., Salgado T.P., Zabal J.M., Adan N.G. (2011). Solitary pancreatic metastasis from renal carcinoma. Gastroenterol. Hepatol..

[151] Masago T., Watanabe T., Nemoto R. (2011). Small renal cell carcinoma with pancreas metastasis: A case report. Hinyokika Kiyo..

[152] Miyao N., Naito S., Ozono S., Shinohara N., Masumori N., Igarashi T., Nakao M., Tsushima T., Senga Y., Horie S. (2011). Late recurrence of renal cell carcinoma: Retrospective and collaborative study of the japanese society of renal cancer. Urology.

[153] Thadani A., Pais S., Savino J. (2011). Metastasis of renal cell carcinoma to the pancreas 13 years postnephrectomy. Gastroenterol. Hepatol..

[154] Watanabe T., Morinaga S., Numata M., Mikayama Y., Tamura S., Tamagawa H., Yamamoto N., Shiozawa M., Ohkawa S., Kameda Y. (2011). Pancreatic resection for metastatic tumors to the pancreas. Gan Kagaku Ryoho.

[155] You D.D., Choi D.W., Choi S.H., Heo J.S., Kim W.S., Ho C.Y., Lee H.G. (2011). Surgical resection of metastasis to the pancreas. J. Korean Surg. Soc..

[156] Alzahrani M., Schmulewitz N., Grewal S., Lucas F., Turner K., McKenzie J., Sussman J., Ahmad S. (2012). Metastases to the pancreas: The experience of. a high volume center and a review of the literature. J. Surg. Oncol..

[157] Çomunoğlu C., Altaca G., Demiralay E., Moray G. (2012). Multiple metastatic renal cell carcinoma isolated to pancreas. Malays. J. Pathol..

[158] Firek P., Richter S., Jaekel J., Brehmer B., Heidenreich A. (2012). Metastasectomy in renal cell cancer after neoadjuvant therapy with multi-tyrosine kinase inhibitors. Urologe.

[159] Gardini A., Morgagni P., Milandri C., Riccobon A., Ridolfi R., La Barba G., Saragoni L., Amadori D., Garcea D. (2012). Pancreatic resection for metastases from renal cancer: Long term outcome after surgery and immunotherapy approach—Single center experience. Hepatogastroenterology.

[160] Hung J.H., Wang S.E., Shyr Y.M., Su C.H., Chen T.H., Wu C.W. (2012). Resection for secondary malignancy of the pancreas. Pancreas..

[161] Katsourakis A., Noussios G., Hadjis I., Alatsakis M., Chatzitheoklitos E. (2012). Late solitary pancreatic metastasis from renal cell carcinoma: A case report. Case Rep. Med..

[162] Yazbek T., Gayet B. (2012). The place of enucleation and enucleo-resection in the treatment of pancreatic metastasis of renal cell carcinoma. JOP.

[163] Zygulska A.L., Wójcik A., Richter P., Krzesiwo K. (2012). Renal carcinoma metachronous metastases to the gall-bladder and pancreas--case report. Pol. Przegl. Chir..

[164] Hata T., Sakata N., Aoki T., Yoshida H., Kanno A., Fujishima F., Motoi F., Masamune A., Shimosegawa T., Unno M. (2013). Repeated pancreatectomy for metachronous duodenal and pancreatic metastases of renal cell carcinoma. Case Rep. Gastroenterol..

[165] Hoshino Y., Shinozaki H., Kimura Y., Masugi Y., Ito H., Terauchi T., Kimatam M., Furukawa J., Kobayashi K., Ogata Y. (2013). Pancreatic metastases from renal cell carcinoma: A case report and literature review of the clinical and radiological characteristics. World J. Surg. Oncol..

[166] Kapoor R., Kumar R., Dey P., Mittal B.R. (2013). A late recurrence of renal cell carcinoma as pancreatic metastases: A rare disease. BMJ Case Rep..

[167] Markinez I., Jiménez R., Ruiz I., Villarreal E., Lizarazu A., Borda N., Arteaga X., Medrano M.Á., Guisasola E., Beguiristain A. (2013). Pancreatic metastases due to renal carcinoma. Our cases and a literature review. Cir. Esp..

[168] Mqirage M., Zabala Egurrola J., Rodríguez J., Pertusa Peña C. (2013). Métastase pancréatique métachrone du cancer du rein: À propos d’un cas. Can. Urol. Assoc. J..

[169] Niess H., Conrad C., Kleespies A., Haas F., Bao Q., Jauch K.W., Graeb C., Bruns C. (2013). Surgery for metastasis to the pancreas: Is it safe and effective?. J. Surg. Oncol..

[170] Simtniece Z., Kirsakmens G., Strumfa I., Vanaga A., Gardovskis J. (2013). Delayed pancreatic metastasis of renal clear cell carcinoma. Acta Chir. Latv..

[171] Yabe N., Murai S., Shimizu H., Kitasato K., Yoshikawa T., Oto I., Nakadai J., Hasegawa H., Kitagawa Y. (2013). A case of pancreatic metastasis from renal cell carcinoma 27 years after nephrectomy. Gan Kagaku Ryoho..

[172] Yoshikawa Y., Murakami M., Shimizu J., Yasuyama A., Watase C., Kubota M., Miyake Y., Matsuura Y., Kim H.M., Hirota M. (2013). A case of partial pancreatectomy for recurrent metastatic renal cell carcinoma in the remnant pancreas after subtotal stomach-preserving pancreaticoduodenectomy. Gan Kagaku Ryoho..

[173] Untsch B.R., Allen P.J. (2014). Pancreatic metastasectomy: The Memorial Sloan-Kettering experience and a review of the literature. J. Surg. Oncol..

[174] Espinoza E., Hassani A., Vaishampayan U., Shi D., Pontes E., Weaver D. (2014). Surgical excision of duodenal/pancreatic metastatic renal cell carcin oma. Front Oncol.

[175] Lauro S., Onesti E.C., Righini R., Carbonetti F., Cremona A., Marchetti P. (2014). A synchronous pancreatic metastasis from renal clear cell carcinoma, with unusual CT characteristics, completely regressed after therapy with sunitinib. Case Rep. Med..

[176] Kimura Y., Keira Y., Imamura M., Ito T., Nobuoka T., Mizuguchi T., Masumori N., Hasegawa T., Hirata K. (2014). Histopathological aspects of pancreatic metastases in renal cell carcinoma: Does the mode of invasion permit limited resections?. Pancreat. Disord. Ther.

[177] Matsuki M., Ichihara K., Matsuda Y., Taguchi K. (2014). Clinical features of six patients with pancreas metastasis from renal cell carcinoma. Hinyokika Kiyo..

[178] Macrì A., Fleres F., Putortì A., Lentini M., Ascenti G., Mastrojeni C. (2014). Relapsed metachronous pancreatic metastasis from renal cell carcinoma (RCC): Report of a case and review of literature. Ann. Ital. Chir..

[179] Minni F., Casadei R., Perence B., Greco V.M., Marrano N., Margiotta A., Marrano D. (2014). Pancreatic metastases: Observations of three cases and review of the literature. Pancreatology..

[180] Moletta L., Milanetto A.C., Vincenzi V., Alaggio R., Pedrazzoli S., Pasquali C. (2014). Pancreatic secondary lesions from renal cell carcinoma. World J. Surg..

[181] Schwarz L., Sauvanet A., Regenet N., Mabrut J.Y., Gigot J.F., Housseau E., Millat B., Ouaissi M., Gayet B., Fuks D. (2014). Long-term survival after pancreatic resection for renal cell carcinoma metastasis. Ann. Surg. Oncol..

[182] Takeshi A., Mitsuhiro I., Hiromitsu A., Naoyuki Y., Taiichiro S., Hiroki S., Takeaki K., Tatsuya S., Futoshi O., Hiroharo S. (2014). Middle segment-preserving pancreatectomy for recurrent metastasis of renal cell carcinoma after pancreatoduodenenctomy: A case report. Case Rep. Surg..

[183] Tosoian J.J., Cameron J.L., Allaf M.E., Hruban R.H., Nahime C.B., Pawlik T.M., Pierorazio P.M., Reddy S., Wolfgang C.L. (2014). Resection of isolated renal cell carcinoma metastases of the pancreas: Outcomes from the Johns Hopkins Hospital. J. Gastrointest. Surg..

[184] Benhaim R., Oussoultzoglou E., Saeedi Y., Mouracade P., Bachellier P., Lang H. (2015). Pancreatic metastasis from clear cell renal cell carcinoma: Outcome of an aggressive approach. Urology.

[185] Chang Y., Liaw C., Chuang C. (2015). The role of surgery in renal cell carcinoma with pancreatic metastasis. Biomed. J..

[186] Gajendra S., Sachdev R., Mohapatra I., Goel R., Goel S. (2015). Metastatic renal cell carcinoma: An unusual cause of bleeding pancreatic mass. J. Clin. Diagn. Res..

[187] Kitade H., Yanagida H., Yamada M., Matsuura T., Yoshioka K., Satoi S., Matsui Y., Kon M. (2015). Pylorus-preserving total pancreatectomy for metastatic renal cell carcinoma: A case report. J. Med. Case Rep..

[188] Kusnierz K., Mrowiec S., Lampe P. (2015). Results of surgical management of renal cell carcinoma metastatic to the pancreas. Contemp. Oncol..

[189] Santoni M., Conti A., Partelli S., Porta C., Sternberg C.N., Procopio G., Bracarda S., Basso U., De Giorgi U., Derosa L. (2015). Surgical resection does not improve survival in patients with renal metastases to the pancreas in the era of tyrosine kinase inhibitors. Ann. Surg. Oncol..

[190] Wiltberger G., Bucher J.N., Kremnzien F., Atanasov G., Schemelzle M., Haum H.M., Bartels M. (2015). Extended resection in pancratic metastases: Feasibility, frequency, and long-term-outcome: A retrospective analysis. BMC Surg..

[191] Yuasa T., Inoshita N., Saiura A., Yamamoto S., Urakami S., Masusa H., Fujii Y., Fukui I., Ishikawa Y., Yonese J. (2015). Clinical outcome of patients with pancreatic metastases from renal cell cancer. BMC Cancer.

[192] Baltazar M.P., Meirinha A., Joao R., Pina M.J., Pinheiro H., Fernandes F., Falcao G., Forte J.P., Carvalho A.M., Vigia E. (2016). Obstructive jaundice as a rare presentation of metastatic renal cell carcinoma—Clinical case and literature review. Acta Urol. Port..

[193] Boussios S., Zerdes J., Batsi O., Papakostas P., Seraj E., Pentheroudakis G., Glantzounis G. (2016). Pancreatic resection for renal cell carcinoma metastasis: An exceptionally rare coexistence. Int. J. Surg. Case Rep..

[194] Dong J., Cong L., Zhang T.P., Zhao Y.P. (2016). Pancreatic metastasis of renal cell carcinoma. Hepatobiliary Pancreat. Dis. Int..

[195] Fikatas P., Klein F., Andreou A., Schmuck R.B., Pratschke J., Bahra M. (2016). Long-term survival after surgical treatment of renal cell carcinoma metastasis within the pancreas. Anticancer Res..

[196] Koga C., Murakami M., Shimizu J., Matsumara T., Kameda C., Kawabata R., Oda N., Hirota M., Yoshikawa M., Morishima H. (2016). A case of multiple pancreatic metastases from renal cell carcinoma diagnosed using EUS-FNA. Gan Kagaku Ryoho.

[197] Miura T., Nakamura N., Ogawa K., Watanabe Y., Yonekura K., Sanada T., Kuwabara H., Goseki N. (2016). Resection of pancreatic metastasis from renal cell carcinoma 21 years after nephrectomy. Gan Kagaku Ryoho.

[198] Nihei K., Sakamoto K., Suzuki S., Mishina T., Otaki M. (2016). A case of pancreatic metastasis of renal cell carcinoma. Gan To Kagaku Ryoho..

[199] Rückert F., Distler M., Ollmann D., Lietzmann A., Birgin E., Teoule P., Grützmann R., Wilhelm T.J. (2016). Retrospective analysis of survival after resection of pancreatic renal cell carcinoma metastases. Int. J. Surg..

[200] Chatzizacharias N.A., Rosich-Medina A., Dajani K., Harper S., Huguet E., Liau S.S., Praseedom R.K., Jah A. (2017). Surgical management of hepato-pancreatic metastasis from renal cell carcinoma. World J. Gastrointest. Oncol..

[201] Garcia-Major Fernandez R.L., Fernandez-Gonzales M. (2017). Diagnosis and treatment of isolated metastases from renal clear cell carcinoma: Report of a case and review of literature. Cr. Cir..

[202] Ko S., Yun S., Kim S., Kim T., Seo H. (2017). Pancreatic resection for renal cell carcinoma metastasis: A case review. Ann. Hepatobiliary Pancreat. Surg..

[203] Lee S.R., Gemenetzis G., Cooper M., Javed A.A., Cameron J.L., Wolfgang C.L., Eckhauser F.E., He J., Weiss M.J. (2017). Long-term outcomes of 98 surgically resected metastatic tumors in the pancreas. Ann. Surg. Oncol..

[204] Shatveryan G.A., Chardarov N.K., Bagmet N.N., Ratnikova N.P., Bedzhanyan A.L., Petrenko K.N., Polishchuk L.O., Karagyozyan G.A. (2017). Isolated pancreatic metastases of renal cell carcinoma. Khirurgiia.

[205] Yagi T., Hashimoto D., Taki K., Yamamura K., Chikamoto A., Ohmuraya M., Beppu T., Baba H. (2017). Surgery for metastatic tumors to the pancreas. Surg. Case Rep..

[206] Zianne M., Takahashi N., Tsujibata A., Miwa K., Goto Y., Matano Y. (2017). Asymptomatic pancreatic metastasis from renal cell carcinoma diagnosed 21 years after nephrectomy. Case Rep. Gastrointest. Med..

[207] Boni A., Cochetti G., Ascani S., Del Zingaro M., Quadrini F., Paladini A., Cocca D., Mearini E. (2018). Robotic treatment of oligometastatic kidney tumor with synchronous pancreatic metastasis: Case report and review of the literature. BMC Surg..

[208] Ito T., Takada R., Omoto S., Tsuda M., Masuda D., Kato H., Matsumoto T., Moriyama I., Okabe Y., Shiomi H. (2018). Analysis of prognostic factors in pancreatic metastasis: A multicentre retrospective analysis. Pancreas.

[209] Kling S.M., Tannouri S., Jiang W., Yeo C.J. (2018). Pancreatic mass in a patient with a history of resected renal cell carcinoma and resected adenocarcinoma of the ampulla of Vater: A case report. J. Pancreat. Cancer.

[210] Limaiem F., Bouraoui S. (2018). Metastasis of renal cell carcinoma to the pancreas 11 years postnephrectomy. Pan. Afr. Med. J..

[211] Madkhali A., Shin S., Song K., Lee J., Hwang D., Paark K., Lee Y., Kim S. (2018). Pancreatectomy for secondary metastasis to the pancreas. Medicine.

[212] Nogueira M., Dias S.C., Silva A.C., Pintob J., Machado J. (2018). Solitary pancreatic renal cell carcinoma metastasis. Autops. Case Rep..

[213] Yamashita H., Toyama H., Terai S., Mukubou H., Shirakawa S., Ishida J., Asakura Y., Shimizu T., Lee D., Tanaka M. (2018). A patient with multiple pancreatic metastases undergoing total pancreatectomy 18 years after renal cell carcinoma resection. Gan Kagaku Ryoho..

[214] Yu Q., Kan F., Ma Z., Wang T., Lin G., Chen B., Zhao W. (2018). CT Diagnosis for metastasis of clear cell renal cell carcinoma to the pancreas: Three case reports. Medicine.

[215] Anderson B., Williams G., Sanford D.E., Lu J., Khan A.S. (2019). A 22-year experience with pancreatic resection for metastatic renal cell carcinoma. HPB.

[216] Ayari Y., Ben Rhouma S., Boussaffa H., Chelly B., Hamza K., Sellami A., Jrad M., Nouira Y. (2019). Metachronous isolated locally advanced pancreatic metastasis from chromophobe renal cell carcinoma. Int. J. Surg. Case Rep..

[217] Brozzetti S., Sterpetti A.V. (2019). Unexpected prolonged survival after extended and emergent resection of pancreatic metastases from renal cell carcinoma. J. Gastrointest. Cancer.

[218] Chon H.K., Choi K.H. (2019). Late metachronous isolated pancreatic metastasis from renal cell carcinoma mimicking a pancreatic neuroendocrine tumor. Turk. J. Gastroenterol..

[219] Endo Y., Noda H., Watanabe F., Kato T., Kakizawa N., Ichida K., Kasahara N., Rikiyama T. (2019). A retrospective analysis of preoperative evaluation and surgical resection for metastatic tumors of the pancreas. Indian J. Surg. Oncol..

[220] Geramizadeh B., Kashkooe A., Nikeghbalian S., Malek-Hosseini S. (2019). Metastatic tumors to the pancreas, a single center study. Arch. Iran. Med..

[221] Glinka J., Sanchez Claria R., Ardiles V., de Santibañes E., Pekolj J., de Santibañes M., Mazza O. (2019). The pancreas as a target of metastasis from renal cell carcinoma: Results of surgical treatment in a single institution. Ann. Hepatobiliary Pancreat. Surg..

[222] Huang Q., Zhou H., Liu C., Jin K., Fan K., Cheng H., Fan Z., Yang C., Liu L., Long J. (2019). Surgical resection for metastatic tumors in the pancreas: A single-center experience and systematic review. Ann. Surg. Oncol..

[223] Jo S., Yang I.S., Song S. (2019). Surgery for metastatic renal cell carcinoma in the pancreatic head: A case report and literature review. Ann. Hepatobiliary Pancreat. Surg..

[224] Ma Y., Yang J., Qin K., Zhou Y., Ying X., Yuan F., Shi M., Jin J., Wang D., Gu J. (2019). Resection of pancreatic metastatic renal cell carcinoma: Experience and long-term survival outcome from a large center in China. Int. J. Clin. Oncol..

[225] Patyutko Y.I., Kotelnikov A.G., Kriger A.G., Prodkuryakov I.S., Galkin G.V., Polyakov A.N., Fainstein I.A. (2019). Metastatic renal cell carcinoma in the pancreas: Experience of surgical treatment. Khirurgiia.

[226] Teranishi R., Hatanaka N., Hara S., Takayama K., Shimura Y., Ohashi T., Osawa H., Sakai K., Yasumasa K., Noro H. (2019). Two cases of pancreatectomy for pancreatic metastasis from renal cell carcinoma. Gan Kagaku Ryoho..

[227] Yamaguchi H., Kimura Y., Nagayama M., Imamura M., Tanaka S., Yoshida E., Fujino H., Machiki T., Miyanishi K., Mizuguchi T. (2019). Central pancreatectomy in portal annular pancreas for metastatic renal cell carcinoma: A case report. World J. Surg. Oncol..

[228] Wakabayashi T., Uchida T., Oyama H., Shiozawa T., Kigawa G., Tanaka K. (2019). A case of laparoscopic distal pancreatectomy for metachronous pancreatic metastasis from renal cell carcinoma. Nihon Rinsho Geka Gakkai Zasshi.

[229] Brozzetti S., Bini S., De Lio N., Lombardo C., Boggi U. (2020). Surgical-only treatment of pancreatic and extra-pancreatic metastases from renal cell carcinoma—quality of life and survival analysis. BMC Surg..

[230] Chikhladze S., Lederer A.K., ·Kühlbrey C.M., ·Hipp J., Sick O., Fichtner-Feigl S., Wittel U.A. (2020). Curative-intent pancreas resection for pancreatic metastases: Surgical and oncological results. Clin. Exper. Metastasis.

[231] Choucair K., Parker N.A., Al-Obaidi A., Alderson J., Truong P. (2020). Solitary, late metastatic recurrence of renal cell carcinoma to the pancreas: A case report. Cureus.

[232] Di Franco G., Gianardi D., Palmeri M., Furbetta N., Guadagni S., Bianchini M., Bonari F., Sbrana A., Vasile E., Pollina L.E. (2020). Pancreatic resections for metastases: A twenty-year experience from a tertiary care center. Eur. J. Surg. Oncol..

[233] Fahlbusch T., Luu A.M., Braumann C., Lukas C., Uhl W., Künzli B.M. (2020). Lipomatous pancreas facilitates late onset of renal cell carcinoma metastases. Acta Chir. Belg..

[234] Janevska V., Shumkovski A., Nikolova D., Asani L., Pandilov S., Karanfilovski V. (2020). Late onset of pancreatic metastases from renal cell carcinoma. A case report. Prilozi.

[235] Milanetto A.C., Morelli L., Di Franco G., David A., Campra D., De Paolis P., Pasquali C. (2020). A plea for surgery in pancreatic metastases from renal cell carcinoma: Indications and outcome from a multicenter surgical experience. J. Clin. Med..

[236] Rupert K., Kural T., Skalický T., Zeithaml J., Hess O., Třeška V. (2020). Clear cell renal carcinoma metastases to the pancreas. Rozhl. Chir..

[237] Schammel J., Schammel C., Schammel D., Trocha S.D. (2020). Renal cell carcinoma metastasis to the pancreas: The aggressive nature of synchronous presentation—Case report and comprehensive review of the literature. SN. Compr. Clin. Med..

[238] Singla N., Xie Z., Zhang Z., Gao M., Yousuf Q., Onabolu O., McKenzie T., Tcheuyap V.T., Ma Y., Choi J. (2020). McKay R, Christie A, Torras RO, Bowman IA, Margulis V, Pedrosa I, Przybycin C, Wang T, Kapur P, Rini B.; Brugarolas JP. Pancreatic tropism of metastatic renal cell carcinoma. JCI. Insight..

[239] Zhang Z.Y., Li X.Y., Bai C.M., Zhou Y., Wu X., Yang A.M., Hua S.R. (2020). The clinicopathologic features and prognostic analysis of pancreatic metastasis from clear cell renal cell carcinoma. Zhonghua Zhong Liu Za Zhi..

[240] Zurimendi G.G., Ibarguren R.L., Castaños D.L., Pereda R.F., Olabarrieta A.U., Casasola-Rodriguez G.G., Egurrola A.Z., Amuza-Echevarria A.A. (2020). Metastasis pancreaticas de tumor primario renal: Presentacion de una serie de casos y revision de la literatura. Arch. Esp. Urol.

[241] Alayyaf N., AlQatari A.A., Altalib A., AlQattan A.S., AlShahrani A.A. (2021). Management of very late pancreatic metastasis of renal cell carcinoma 8 years after radical nephrectomy: A report of a rare case. Am. J. Case Rep.

[242] Bauschke A., Altendorf-Hofmann A., Deeb A.A., Kissler H., Tautenhahn H.M., Settmacher U. (2021). Chirurgische Therapie von Leber und Pankreasmetastasen von Nierenzellkarzinomen. Chirurg.

[243] Blanco-Fernández G., Fondevila-Campo C., Sanjuanbenito A., Fabregat-Prous J., Secanella-Medayo L., Rotellar-Sastre F., Pardo-Sanchez F., Prieto-Calvo M., Marin-Ortega H., Sanchez-Cabus S. (2022). Pancreatic metastases from renal cell carcinoma. Postoperative outcome after surgical treatment in a Spanish multicenter study. Eur. J. Surg. Oncol..

[244] Ksontini F., Khrouf S., Kacem S., Hadda A., Magherbi H., Chaker Y., Ayadi M., Ben Safta Z. (2021). Pancreatic metastasis of renal cell carcinoma: A surgical indication for a disseminated disease. Case Rep. Med..

[245] Malleo G., Salvia R., Maggino L., Marchegiani G., D’Angelica M., DeMatteo R., Kingham P., Pulvirenti A., Sereni E., Jarnagin W.R. (2021). Long-term outcomes after surgical resection of pancreatic metastases from renal clear-cell carcinoma. Ann. Surg. Oncol..

[246] Matsui S., Ono H., Asano D., Ishikawa Y., Ueda H., Akahoshi K., Ogawa K., Kudo A., Tanaka S., Tanabe M. (2021). Pancreatic metastasis from renal cell carcinoma presenting as gastrointestinal hemorrhage: A case report. J. Surg. Case Rep..

[247] Novotny A., Sell E., Mehrotra S. (2021). Metastatic tumors to the pancreas, a 12-year single institution review. Diagn. Cytopathol..

[248] Piskorz Ł., Mitura K., Olejniczak W., Misiak P., Jablonski S. (2021). Atypical locations of renal cell carcinoma metastases to the pancreas and duodenum. Res. Rep. Urol..

[249] Yamada Y., Sakai A., Abe S., Gonda M., Kobayashi T., Masusa A., Shiomi H., Sahirakawa S., Toyama H., Hyodo T. (2021). Pancreatic metastasis of renal cell carcinoma filling into the duct of Santorini. Clin. J. Gastroenterol..

[250] Yamawaki M., Takano Y., Noda J., Azami T., Kobayashi T., Niiya F., Maruoka T., Nagashama M. (2022). A case of hemobilia caused by pancreatic metastasis of renal cell carcinoma treated with a covered metallic stent. Clin. J. Gastroenterol..

[251] Cardoso D., Rosales A., Thiel D.D., Asbun H., Stauffer J.A. (2022). Pancreatic metastasectomy of renal cell carcinoma: A single institution experience. Can. J. Urol..

[252] Itamoto S., Abe T., Oshita A., Hanada K., Nakahara M., Noriyuki T. (2022). Repeat pancreatic resection for metachronous pancreatic metastasis from renal cell carcinoma: A case report. Int. J. Surg. Case Rep..

[253] Liang X.K., Li L.J., He Y.M., Xu Z.F. (2022). Misdiagnosis of pancreatic metastasis from renal cell carcinoma: A case report. World J. Clin. Cases.

[254] Yano R., Yokota T., Morita M., Amano M., Ochi H., Azemoto N., Mashiba T., Joko K. (2022). A case of metastasis from renal cell carcinoma to ectopic pancreas diagnosed after resection. Intern. Med..

[255] Sbitti Y., Debbagh A., Slimani K., Mahi M., Errihani H., Ichou M. (2018). When tyrosine kinase inhibitor sunitinib can be discontinued in metastatic renal cell carcinoma to pancreas: A case report. J. Med. Case Rep..

[256] Chara L., Rodriguez B., Holgado E., Ramirez N., Fernandez-Rañada I., Mohedano N., Arcediano A., Garcia I., Cassinello J. (2011). An unusual metastatic renal cell carcinoma with maintained complete response to sunitinib treatment. Case Rep. Oncol..

[257] Medioni J., Choueiri T.K., Zinzindohoué F., Cho D., Fournier L., Oudard S. (2009). Response of renal cell carcinoma pancreatic metastasis to sunitinib treatment: A retrospective analysis. J. Urol..

[258] Negishi T., Furubayashi N., Nakagawa T., Nishiyama N., Kitamura H., Hori Y., Kuroiwa K., Son Y., Seki N., Tomoda T. (2021). Site specific response to Nivolumab in renal cell carcinoma. Anticancer Res..

[259] Reddy S., Edil B.H., Cameron J.L., Pawlik T.M., Herman J.M., Gilson M.M., Campbell K.A., Schulick R.D., Ahuja N., Wolfgang C.L. (2008). Pancreatic resection of isolated metastases from nonpancreatic primary cancers. Ann. Surg. Oncol..

[260] Iacovelli R., Lanoy E., Albiges L., Escudier B. (2012). Tumour burden is an independent prognostic factor in metastatic renal cell carcinoma. BJU Int..

[261] Grassi P., Verzoni E., Mariani L., De Braud F., Coppa J., Mazzaferro V., Procopio G. (2013). Prognostic role of pancreatic metastases from renal cell carcinoma: Results from an Italian center. Clin. Genitourin. Cancer.

[262] Shaya J.A., Lin X., Weise N., Cabal A., Panian J., Derweesh I.H., McKay R.R. (2021). Prognostic significance of pancreatic metastases in patients with advanced renal cell carcinoma treated with systemic therapy. Clin. Genitourin. Cancer.

[263] Shin T.J., Song C., Jeong C.W., Kwak C., Seo S., Kang M., Chung J., Hong S.H., Hwang E.C., Park J.Y. (2021). Metastatic renal cell carcinoma to the pancreas: Clinical features and treatment outcome. J. Surg. Oncol..

[264] Dudani S., de Velasco G., Wells J.C., Gan C.L., Donskov F., Porta C., Pasini F., Lee J.L., Hansen A., Bjarnason G.A. (2021). Evaluation of clear cell, papillary, and chromophobe renal cell carcinoma metastasis sites and association with survival. JAMA Netw. Open.

[265] Grassi P., Doucet L., Giglione P., Grünwald V., Melichar B., Galli L., De Giorgi U., Sabbatini R., Ortega C., Santoni M. (2016). Clinical impact of pancreatic metastases from renal cell carcinoma: A multicenter retrospective analysis. PLoS ONE.

[266] Chrom P., Stec R., Bodnar L., Szczylik C. (2018). Prognostic significance of pancreatic metastases from renal cell carcinoma in patients treated with tyrosine kinase inhibitors. Anticancer Res..

[267] Kalra S., Atkinson B.J., Matrana M.R., Matin S.F., Wood C.G., Karam J.A., Tamboli P., Sircar K., Rao P., Corn P.G. (2016). Prognosis of patients with metastatic renal cell carcinoma and pancreatic metastases. BJU Int..

[268] The Cancer Genome Atlas Research Network (2013). Comprehensive molecular characterization of clear renal cell carcinoma. Nature.

[269] Jonasch E. (2018). Updates to the management of kidney cancers. J. Natl. Compr. Canc. Netw..

[270] Mitchell T.J., Rossi S.H., Klatte T., Stewart G.D. (2018). Genomics and clinical correlates of renal cell carcinoma. W. J. Urol..

[271] Carlo M.I., Manley B., Patil S., Woo K.M., Coskey D.T., Redzematovic A., Arcila M., Ladanyi M., Lee W., Chen Y.B. (2017). Genomic alterations and outcomes with VEGF-targeted therapy in patients with clear cell renal cell carcinoma. Kidney Cancer.

[272] Voss M.H., Reising A., Cheng Y., Patel P., Marker M., Kuo F., Chan T.A., Choueiri T.K., Hsieh J.J., Hakimi A.A. (2018). Genomically annotated risk model for advanced renal-cell carcinoma: A retrospective cohort study. Lancet Oncol..

[273] Turajlic S., Xu H., Litchfield K., Rowan A., Chambers T., Lopez J.I., Nicol D., O’Brien T., Larkin J., Horswell S. (2018). Tracking cancer evolution reveals constrained routes to metastases: TRACERx Renal. Cell.

[274] Meacci E., Nachira D., Zanfrini E., Evangelista J., Triumbari E.K.A., Congedo M.T., Petracca Ciavarella L., Chiappetta M., Vita M.L., Schinzari G. (2021). Prognostic factors affecting survival after pulmonary resection of metastatic renal cell carcinoma: A multicenter experience. Cancers.

[275] Saricam M. (2020). Factors Affecting Long-Term Survival Following Pulmonary Metastasectomy of Renal Cell Carcinoma. Urol. Oncol..

[276] Zhao Y., Li J., Li C., Fan J., Liu L. (2017). Prognostic factors for overall survival after lung metastasectomy in renal cell cancer patients: A systematic review and meta-analysis. World J. Surg..

[277] Macherey S., Kauffmann C., Heidenreich A., Doerr F., Wahlers T., Hekmat K. (2017). Pulmonary metastasectomy in renal cell carcinoma. Urologe A..

[278] Piltz S., Meimarakis G., Wichmann M.W., Hatz R., Schildberg F.W., Fuerst H. (2002). Long-term results after pulmonary resection of renal cell carcinoma metastases. Ann. Thorac. Surg..

[279] Hau H.M., Thalmann F., Lübbert C., Morgul M.H., Schmelzle M., Atanasov G., Benzing C., Lange U., Ascherl R., Ganzer R. (2016). The value of hepatic resection in metastasicrenal cancer in the era of tyrosinkinase inhibitor therapy. BMC Surg..

[280] Aloia T.A., Adam R., Azoulay D., Bismuth H., Castaing D. (2006). Outcome following hepatic resection of metastatic renal tumors: The Paul Brousse Hospital experience. HPB.

[281] Hamada S., Ito K., Kuroda K., Sato A., Asakuma J., Horiguchi A., Seguchi K., Asano T. (2015). Clinical characteristics and prognosis of patients with renal cell carcinoma and liver metastasis. Mol. Clin. Oncol..

[282] Ruys A.T., Tanis P.J., Nagtegaal I.D., van Duijvendijk P., Verhoef C., Porte R.J., van Gulik T.M. (2011). Surgical treatment of renal cell cancer liver metastases: A population-based study. Ann. Surg. Oncol..

[283] Cheng K.C., Yip A.S.M. (2022). Prognostic factors of survival and a new scoring system for liver resection of colorectal liver metastasis. World J. Hepatol..

[284] Villard C., Abdelrafee A., Habib M., Ndegwa N., Jorns C., Sparrelid E., Allard M.A., Adam R. (2022). Prediction of survival in patients with colorectal liver metastases- development and validation of a prognostic score model. Eur. J. Surg. Oncol..

[285] Acciuffi S., Meyer F., Bauschke A., Croner R., Settmacher U., Altendorf-Hofmann A. (2022). Solitary colorectal liver metastasis: Overview of treatment strategies and role of prognostic factors. J. Cancer Res. Clin. Oncol..

[286] Fromer M.W., Scoggins C.R., Egger M.E., Philips P., McMasters K.M., Martin R.C.G. (2022). Preventing futile liver resection: A risk-based approach to surgical selection in major hepatectomy for colorectal cancer. Ann. Surg. Oncol..

[287] Viganò L., Gentile D., Galvanin J., Corleone P., Costa G., Cimino M., Procopio F., Torzilli G. (2022). Very early recurrence after liver resection for colorectal metastases: Incidence, risk factors, and prognostic impact. J. Gastrointest. Surg..

[288] Ren W., Sell N.M., Ferrone C.R., Tanabe K.K., Lillemoe K.D., Qadan M. (2021). Size of the largest colorectal liver metastasis Is an independent prognostic factor in the neoadjuvant setting. J. Surg. Res..

[289] Moro A., Mehta R., Tsilimigras D.I., Sahara K., Paredes A.Z., Bagante F., Guglielmi A., Alexandrescu S., Poultsides G.A., Sasaki K. (2020). Prognostic factors differ according to KRAS mutational status: A classification and regression tree model to define prognostic groups after hepatectomy for colorectal liver metastasis. Surgery.

[290] Carvajal C., Facundo H., Puerto P., Carreño J., Beltrán R. (2022). Lung metastasectomy from colorectal cancer, 10-year experience in a South American cancer center. Front. Surg..

[291] Gössling G.C.L., Chedid M.F., Pereira F.S., da Silva R.K., Andrade L.B., Peruzzo N., Saueressig M.G., Schwartsmann G., Parikh A.R. (2021). Outcomes and prognostic factors of patients with metastatic colorectal cancer who underwent pulmonary metastasectomy with curative intent: A brazilian experience. Oncologist.

[292] Okumura T., Boku N., Hishida T., Ohde Y., Sakao Y., Yoshiya K., Higashiyama M., Hyodo I., Mori K., Kondo H. (2017). Surgical outcome and prognostic stratification for pulmonary metastasis from colorectal cancer. Ann. Thorac. Surg..

[293] Shimizu K., Ohtaki Y., Okumura T., Boku N., Horio H., Takenoyama M., Yamashita M., Hyodo I., Mori K., Kondo H. (2019). Outcomes and prognostic factors after pulmonary metastasectomy in patients with colorectal cancer with previously resected hepatic metastases. J. Thorac. Cardiovasc. Surg..

[294] Brozzetti S., Carati M., Sterpetti A. Systematic review and metanalysis of clinical outcomes after enucleation of pancreatic metastases from renal cell carcinoma. Dig. Surg..

[295] Saitoh H., Kobayashi N., Yochida K., Suwata L., Uchijima Y., Nakame Y. (1997). Possible metastatic routes via portocaval shunts in renal adenocarcinoma with liver metastasis. Urology.

[296] Lore J., Madden J., Gerold F. (1958). Pre-exisiting portocaval shunts: A hypothesis for bizarre metastases of some carcinomas. Cancer.

[297] Sellner F. (2018). Isolated pancreatic metastases from renal cell carcinoma: An outcome of a special metastatic pathway or of a specific tumor cell selection?. Clin. Exp. Metastasis.

[298] Jaen-Torrejimeno I., Rojas-Holguin A., Lopez-Querra D., Ramia J.M., Blanco-Fernandez Q. (2020). Pancreatic resection for metastatic renal cell carcinoma. A systematic review. HPB.

[299] Sellner F. (2020). Isolated pancreatic metastases of renal cell carcinoma—A paradigm of a seed and soil mechanism: A literature analysis of 1034 observations. Front. Oncol..

[300] Sellner F., Thalhammer S., Klimpfinger M. (2022). Isolated pancreatic metastases of renal cell cancer: Genetics and epigenetics of an unusual tumour entity. Cancers.

[301] Paget S. (1889). The distribution of secondary growths in cancer of the breast. Lancet.

[302] Akhtar M., Haider A., Rashid S.M., Al-Naber A.D. (2019). Paget’s "Seed and Soil" theory of cancer metastasis: An idea whose time has come. Adv. Anat. Pathol..

[303] Chambers A., Varghese H., Nadkarni K., MacDonald I., Groom A. (2001). Critical steps in hematogenous metastasis: An overview. Surg. Oncol. Clin. N. Am..

[304] Hunter K. (2004). Host genetics and tumour metastasis. Br. J. Cancer.

[305] Chiang S.P., Cabrera R.M., Segall J.E. (2016). Tumor cell intravasation. Am. J. Physiol. Cell Physiol..

[306] Obenauf A.C., Massague J. (2015). Surviving at a distance: Organ-specific metastasis. Trends Cancer.

[307] Summers M.A., McDonald M.M., Croucher P.I. (2020). Cancer cell dormancy in metastasis. Cold Spring Harb. Perspect. Med..

[308] Endo H., Inoue M. (2019). Dormancy in cancer. Cancer Sci..

[309] Liu Q., Zhang H., Jiang X., Qian C., Liu Z., Luo D. (2017). Factors involved in cancer metastases: A better understanding to “seed and soil” hypothesis. Mol. Cancer.

[310] Gao Y., Bado I., Wang H., Zhang W., Rosen J.M., Zhang X.H. (2019). Metastasis organotropism: Redefining the congenial soil. Dev. Cell.

[311] Nan X., Wang J., Liu H.N., Wong S.T., Zhao H. (2019). Epithelial-mesenchymal plasticity in organotropism metastasis and tumor immune escape. J. Clin. Med..

[312] Wang Y., Ding Y., Guo N., Wang S. (2019). MDSCs: Key criminals of tumor pre-metastatic niche formation. Front. Immunol..

[313] Wang H., Pan J., Barsky L., Jacob J.C., Zheng Y., Gao C., Wang S., Zhu W., Sun H., Lu L. (2021). Characteristics of pre-metastatic niche: The landscape of molecular and cellular pathways. Mol. Biomed..

[314] Kaplan R.N., Riba R.D., Zacharoulis S., Bramley A.H., Vincent L., Costa C., MacDonald D.D., Jin D.K., Shido K., Kerns S.A. (2005). VEGFR1-positive haematopoietic bone marrow progenitors initiate the pre-metastatic niche. Nature.

[315] Sceneay J., Smyth M., Möller A. (2013). The pre-metastatic niche: Finding common ground. Cancer Metastasis Rev..

[316] Liu Y., Cao X. (2016). Characteristics and significance of the pre-metastatic niche. Cancer Cell..

[317] Talmadge J.E., Fidler I.J. (2010). AACR centennial series: The biology of cancer metastasis: Historical perspective. Cancer Res..

[318] Grange C., Brossa A., Bussolatti B. (2019). Extracellular vesicles and carried miRNAs in the progression of renal cell carcinoma. Int. J. Mol. Sci..

[319] Gai C., Pomatto M.A., Grange C., Deregibus M.C., Camussi G. (2019). Extracellular vesicles in onco-nephrology. Exp. Mol. Med..

[320] Wortzel I., Dror S., Kenific C.M., Lyden D. (2019). Exosome-mediated metastasis: Communication from a distance. Dev. Cell..

[321] Deguchi A., Maru Y. (2022). Inflammation-associated premetastatic niche formation. Inflamm. Regen..

[322] Grange C., Tapparo M., Collino F., Vitillo L., Damasco C., Deregibus M.C., Tetta C., Bussolati B., Camussi G. (2011). Microvesicles released from human renal cancer stem cells stimulate angiogensis and formation of lung premetastatic niche. Cancer Res..

[323] Walenkamp A.M., Lapa C., Herrmann K., Wester H.J. (2017). CXCR4 ligands: The next big hit?. J. Nucl. Med..

[324] DiNatale A., Castelli M.S., Nash B., Meucci O., Fatatis A. (2022). Regulation of tumor and metastasis initiation by chemokine receptors. J. Cancer.

[325] Schild T., Low V., Blenis J., Gomes A.P. (2018). Unique metabolic adaptations dictate distal organ-specific metastatic colonization. Cancer Cell..

[326] Ebert D., Haller R.G., Walton M.E. (2003). Energy contribution of octanoate to intact rat brain metabolism measured by 13C nuclear magnetic resonance spectroscopy. J. Neurosci..

[327] Da Silva R.P., Nissim I., Brosnan J.T. (2009). Creatine synthesis: Hepatic metabolism of guanidinacetate and creatine in the rat in vitro and in vivo. Am. J. Physiol. Endocrinol. Metab..

[328] Mashimo T., Pichumani K., Vemireddy V., Hatanpaa K.J., Singh D.K., Sirasanagandla S., Nannepaga S., Piccirillo S.G., Kovacs Z., Foong C. (2014). Acetate is a bioenergetic substrate for human glioblastoma and brain metastases. Cell.

[329] Chen J., Lee H.J., Wu X., Huo L., Kim S.J., Xu L., Wang Y., He J., Bollu L.R., Gao G. (2015). Gain of glucose-independent growth upon metastasis of breast cancer cells to the brain. Cancer Res..

[330] Wang C., Luo D. (2021). The metabolic adaptation mechanism of metastatic organotropism. Exp. Hematol. Oncol..

[331] Bloom H.J. (1973). Hormone induced and spontaneous regression of metastatic renal cancer. Cancer.

[332] Snow R.M., Schellhammer P.F. (1982). Spontaneous regression of metastatic renal cell carcinoma. Urology.

[333] Elhilali M.M., Gleave M., Fradet Y., Venner D., Saad F., Klotz L., Moore R., Paton E. (2000). Placebo-associated remissions in a multicentre, randomized, double-blind trial of interferon γ-1b for the treatment of metastatic renal cell carcinoma. BJU. Int..

[334] Rini B.I., Battle D., Figlin R.A., George D.J., Hammers H., Hutson T., Jonasch E., Joseph R.W., McDermott D.F., Motzer R.J. (2019). The society for immunotherapy of cancer consensus statement on immunotherapy for the treatment of advanced renal cell carcinoma (RCC). J. Immunother. Cancer.

[335] Flippot R., Escudier B., Albiges L. (2018). Immune checkpoint inhibitors: Toward new paradigms in renal cell carcinoma. Drugs.

[336] Chang A.J., Zhao L., Zhu Z., Boulanger K., Xiao H., Wakefield M.R., Bai Q., Fang Y. (2019). The past, present and future of immunotherapy for metastatic renal cell carcinoma. Anticancer Res..

[337] Wang T., Lu R., Kapur P., Jaiswal B.S., Hannan R., Zhang Z., Pedrosa I., Luke J.J., Zhang H., Goldstein L.D. (2018). An empirical approach leveraging tumorgrafts to dissect the tumor microenvironment in renal cell carcinoma identifies missing link to prognostic inflammatory factors. Cancer Discov..

[338] Laruelle A., Manini C., Iñarra E., López J.I. (2021). Metastasis, an example of evolvability. Cancers.

